# Inhibition of CHI3L1 attenuates excessive autophagy in intestinal epithelial cells to reduce the severity of necrotizing enterocolitis

**DOI:** 10.1038/s41420-025-02443-7

**Published:** 2025-04-05

**Authors:** Yihui Li, Wenqiang Sun, Xinyun Jin, Huiwen Li, Xue Liu, Jingtao Bian, Xueping Zhu

**Affiliations:** 1 Department of Neonatology, Children’s Hospital of Soochow University, Suzhou, China; 2 Suzhou Medical College, Soochow University, Suzhou, China

**Keywords:** Infant necrotizing enterocolitis, Paediatric research

## Abstract

Neonatal necrotizing enterocolitis (NEC) is a devastating intestinal disease that primarily affects preterm infants. Unfortunately, no specific treatment for NEC is currently available, making it crucial to further investigate its underlying mechanisms. In this study, we aimed to identify the key target gene, CHI3L1, which was significantly upregulated in the intestinal tissues of both affected children and model mice from the GEO database. CHI3L1 is known to play important roles in inflammatory and immune responses, as well as in tissue damage and repair, all of which are closely associated with the development of NEC. We conducted validations at both the cellular and animal levels, demonstrating that the inhibition or knockdown of CHI3L1 significantly reduced the severity of NEC. Mechanistic investigations revealed that the knockdown of CHI3L1 inhibited the PI3K-Akt-FoxO1 signalling pathway, alleviating excessive autophagy in intestinal epithelial cells and subsequently reducing injury and inflammatory responses. Clinical studies have revealed that elevated serum CHI3L1 expression in paediatric patients is associated with both the occurrence and severity of necrotising enterocolitis NEC, demonstrating positive correlations with the Duke Abdominal Assessment Scale (DAAS), C-reactive protein (CRP), procalcitonin (PCT), red cell distribution width (RDW), and lactate dehydrogenase (LDH) levels. In conclusion, our findings confirmed a close relationship between CHI3L1 and the occurrence and severity of NEC, suggesting that it may mitigate inflammatory responses and tissue damage by alleviating excessive autophagy in intestinal epithelial cells. Therefore, targeting CHI3L1 may be an effective strategy to combat NEC.

## Introduction

Necrotizing enterocolitis (NEC) is a devastating inflammatory disease of the intestinal tract, commonly observed in preterm infants [1]. With advancements in perinatal medicine and neonatal critical care, the survival rate of preterm infants has significantly improved; however, the incidence of NEC remains high, with an overall mortality rate of 20–30% [2, 3]. While the pathogenesis of NEC is not yet fully understood, researchers believe that perinatal factors impact the immature intestinal tract of preterm infants, leading to an excessive inflammatory response and tissue damage to the intestinal barrier [4–6]. Currently, no specific treatment for NEC and its management primarily involves conservative surgical approaches. Unfortunately, children who undergo surgical treatment often experience long-term growth and neurodevelopmental delays [7, 8]. Therefore, exploring the pathogenesis of NEC and identifying new specific targets for its prevention and treatment are of great significance.

RNA sequencing is an essential method for analysing the entire transcriptome, focusing on transcript identification and gene expression quantification. This technique offers a broader detection range, greater reproducibility, and more reliable analytical results [9, 10]. It has been extensively utilised in the investigation of disease-specific mechanisms underlying NEC [11, 12]. Significant differences exist in the responses to intestinal injury between humans and mice, despite the increasingly detailed insights gained from NEC mouse models. These disparities may contribute to the failure of many therapies that have shown promise in preclinical animal studies to be successfully translated into clinical practice [13, 14]. Therefore, identifying the shared mechanisms between necrotic intestinal tissue in mouse models of NEC and human children affected by NEC is crucial for achieving successful clinical translation.

In this study, we integrated transcriptomic data from the intestinal tissues of children with NEC and animal models to identify the differentially expressed target gene CHI3L1, which was significantly upregulated in both human and mouse transcriptomic datasets. Based on the biological functions of this gene, we hypothesised that CHI3L1 may play a critical role in NEC pathogenesis. Consequently, we initiated investigations at the cellular, animal, and clinical levels to explore the role of CHI3L1 in mediating excessive inflammatory responses and injury to the intestinal epithelium, as well as the signalling pathways that may be involved. These findings offer new insights and potential targets for interventions for the prevention and treatment of NEC.

## Results

### CHI3L1 is expressed at high levels in children with NEC and animal models

**Fig. 1:**
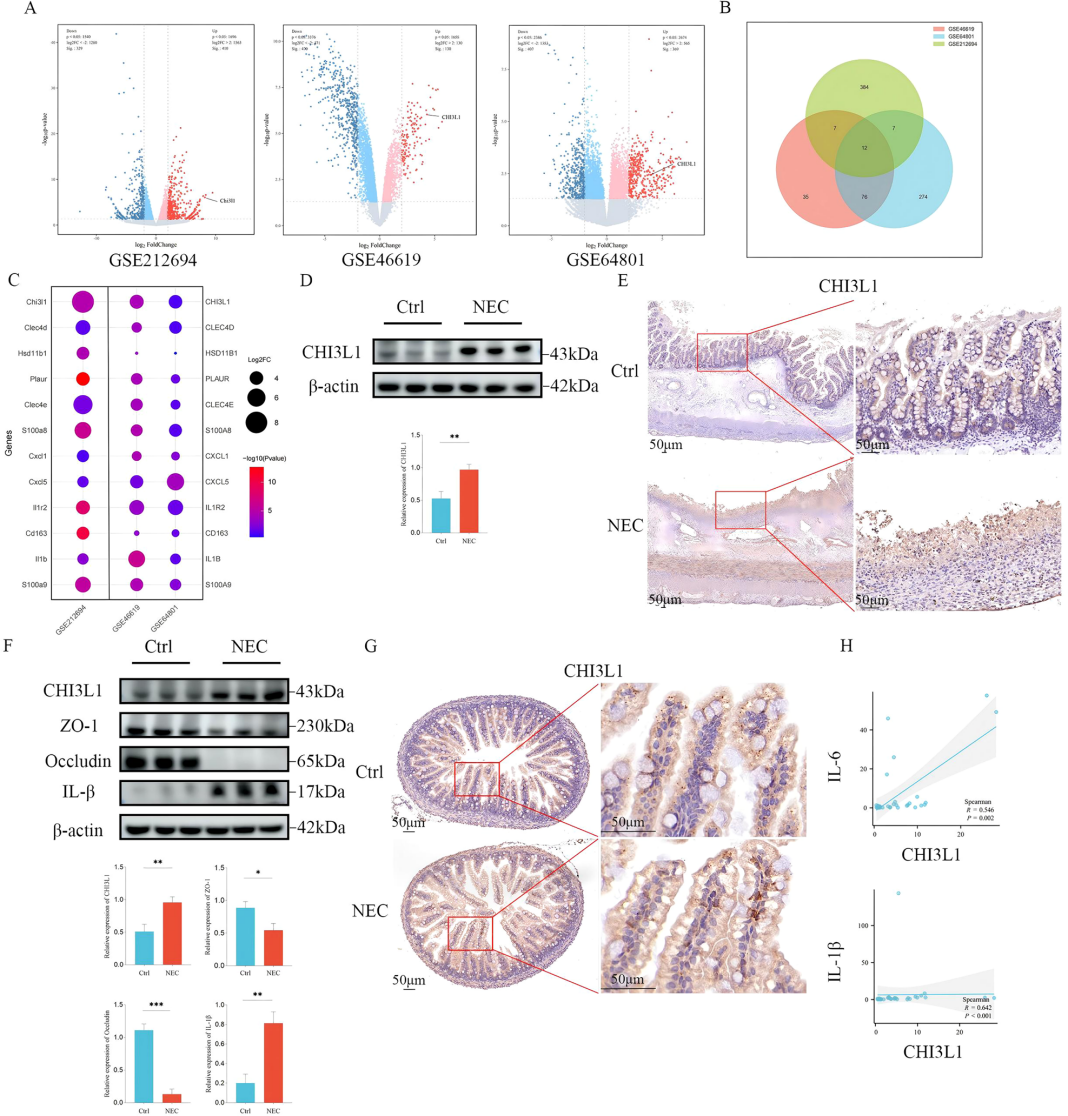
Screening for the high expression of CHI3L1, a gene shared by children with NEC and animal models of NEC. **A**–**C** Analysis of co-expressed genes in human and mouse NEC using data from the GEO database, which revealed significant changes in CHI3L1. **D** Western blot analysis showing the expression of CHI3L1 in clinically collected intestinal tissues from patients with NEC and controls. **E** Immunohistochemical analysis of CHI3L1 expression in representative images from patients with NEC and controls. Scale bar = 50 μm. **F** Western blot showing the expression of CHI3L1, ZO-1, occludin, and IL-1β proteins in control mice and NEC mice. **G** Representative immunohistochemical images of CHI3L1 in NEC mice and controls. **H** Correlation of CHI3L1 with the inflammatory factors IL-1β and IL-6. Scale bar = 50 μm. Data are expressed as mean ± SD (*p < 0.05, **p < 0.01, and ***p < 0.001).

Differential gene identification was performed on NEC datasets (GSE46619, GSE64801, and GSE212694), revealing 739 differentially expressed genes (DEGs) in GSE212694 (410 upregulated and 329 downregulated), 600 DEGs in GSE46619 (130 upregulated and 470 downregulated), and 776 DEGs in GSE64801 (369 upregulated and 407 downregulated) (Fig. 1A). In total, 795 upregulated DEGs were identified across the three datasets, with 12 common upregulated DEGs shared among them (Fig. 1B). Among these, CHI3L1 was significantly expressed in the NEC group across all three datasets (Fig. 1C), whereas it was almost undetectable in the control group. A literature review indicated that CHI3L1 exhibits strong biological activity, regulating various pathophysiological processes and playing a key role in inflammation, immune response, tissue damage, and repair [15, 16]. Therefore, we hypothesised that CHI3L1 is crucial for NEC onset and progression. Western blot analysis demonstrated that freshly collected intestinal tissues from children with NEC exhibited higher CHI3L1 protein expression than those from controls (*p* < 0.05; Fig. 1D). IHC of intestinal tissues indicated that CHI3L1 expression was significantly elevated in the epithelial layer of the small intestine (Fig. 1E). We also confirmed that CHI3L1 levels were significantly increased in the NEC animal model, coinciding with a notable decrease in levels of the barrier proteins ZO-1 and occludin and a significant elevation in levels of IL-1β *(p* < 0.05, Fig. 1F). IHC analysis of animal intestinal tissues further demonstrated that CHI3L1 was highly expressed in the epithelial layer of the NEC group, whereas it was barely detectable in the control group (Fig. 1G). Additionally, CHI3L1 was found to be directly correlated with the inflammatory response [17], and Spearman correlation analysis revealed a positive correlation between CHI3L1 levels and IL-1β (*R* = 0.642, *p* < 0.01) and IL-6 (*R* = 0.546, *p* = 0.002) levels (Fig. 1H). In conclusion, the high expression of CHI3L1 in the intestinal tissues of patients with NEC suggests that it may play a significant role in NEC development.

### Inhibition of CHI3L1 reduces the severity of NEC in vivo

**Fig. 2:**
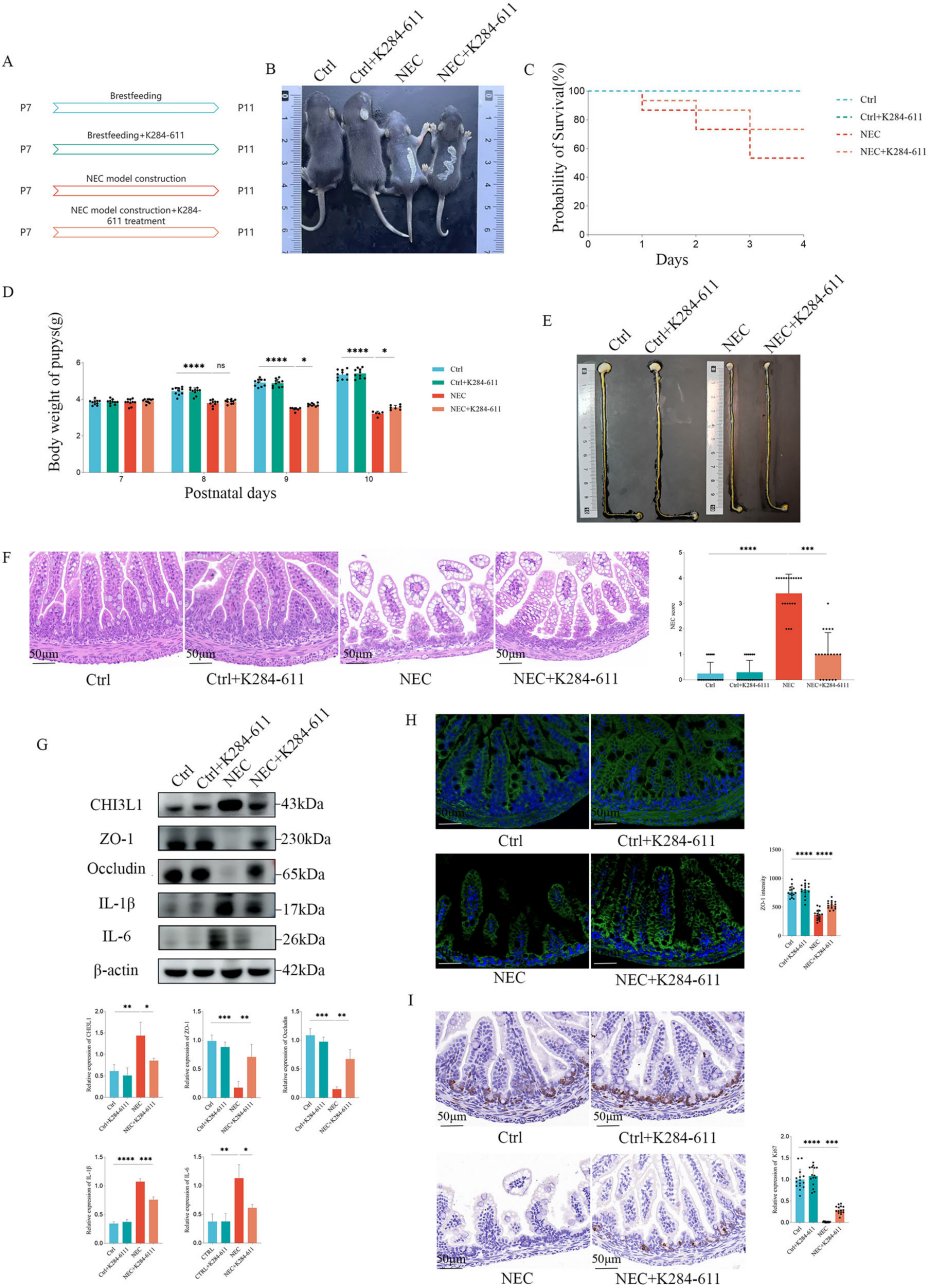
In vivo inhibition of CHI3L1 reduces the severity of NEC. **A** Four groups of mice were established: Ctrl, Ctrl+K284-611, NEC, and NEC + K284-611. **B**–**E** Body size, survival curves, changes in body weight, and intestinal gross morphology of the different groups of mice. **F** HE-stained intestinal sections with representative images from each group. **G** Western blot analysis showing the expression of CHI3L1, ZO-1, Occludin, IL-1β, and IL-6 proteins in the different groups of mice. **H** Representative immunofluorescence images of ZO-1 in the various groups. **I** Representative immunohistochemical images of Ki67 in the different groups. Scale bar = 50 μm. Data are expressed as mean ± SD (*p < 0.05, **p < 0.01, ***p < 0.001, and ****p < 0.0001).

To elucidate the role of CHI3L1 in NEC, we exogenously supplemented the NEC mouse model with the CHI3L1 inhibitor K284-611 (Fig. 2A). During the modelling process, the survival rate of mice in the inhibitor group was significantly higher than that in the NEC group, and NEC mice showed notably reduced weight loss (Fig. 2B–D). Comparison of the intestinal tissues among the groups after modelling revealed that the intestinal length in the NEC group was shorter than that in both the control and control + K284-611 groups. However, the intestines of the NEC + K284-611 group were longer than those of the NEC group (Fig. 2E). HE staining of 1-cm segments of intestinal tissue from the upper ileum showed that the intestinal villi in the control and control + K284-611 groups were regularly and neatly arranged, with the submucosal layer closely connected to the lamina propria. In contrast, the intestinal structure of the NEC group was compromised, resulting in severe villous damage. Importantly, the integrity of the intestinal villi was restored following inhibition of CHI3L1 expression (Fig. 2F). Western blot of intestinal tissues indicated that the levels of barrier proteins ZO-1 and occludin were significantly lower in the NEC group than in the control and control + K284-611 groups. Additionally, levels of the inflammatory factors IL-1β and IL-6 were significantly elevated in the NEC group. After inhibiting CHI3L1, the expression levels of ZO-1 and occludin significantly increased, whereas IL-1β and IL-6 levels significantly decreased (Fig. 2G). Immunofluorescence analysis demonstrated a marked increase in ZO-1 expression in intestinal tissues following CHI3L1 inhibition (Fig. 2H). Furthermore, intestinal IHC revealed a significant decrease in Ki67 expression in the NEC group, which was notably elevated after the inhibition of CHI3L1 expression (Fig. 2I), suggesting that the inhibition of CHI3L1 promoted intestinal cell proliferation in mice with NEC. Collectively, these results indicated that inhibition of CHI3L1 in vivo can reduce the severity of NEC.

### Knockdown of CHI3L1 promotes proliferation and migration and reduces apoptosis in an in vitro model of NEC

**Fig. 3:**
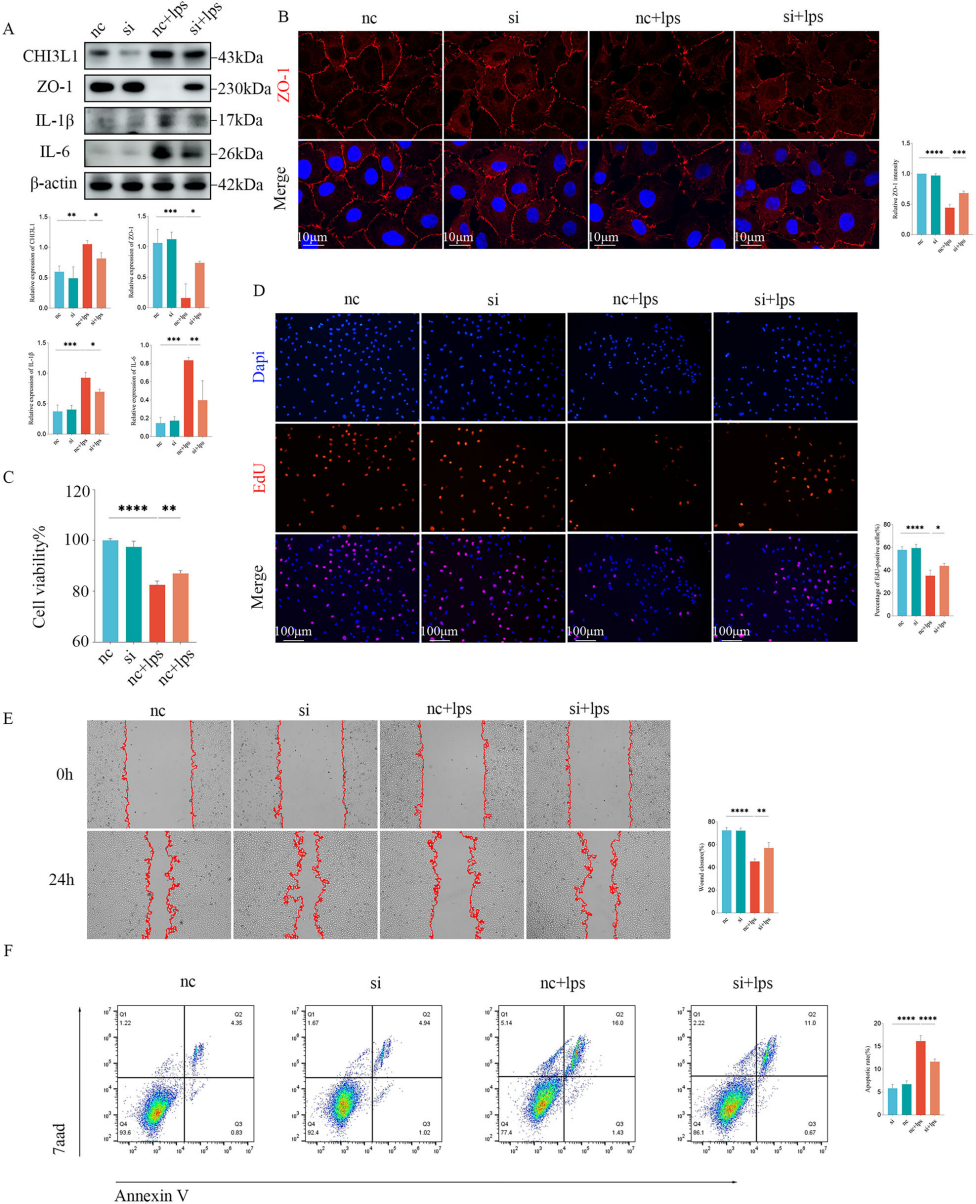
Knockdown of CHI3L1 inhibits apoptosis and inflammatory response while promoting proliferation, migration, and barrier repair in NEC intestinal epithelial cells. **A** Western blot analysis showing the expression of CHI3L1, ZO-1, IL-1β, and IL-6 proteins across different groups of cells. **B** Representative immunofluorescence images of ZO-1 in IEC-6 cells with and without CHI3L1 knockdown. Scale bar = 10 μm. **C**, **D** CCK-8 and EdU assays were used to assess the effect of CHI3L1 knockdown on cell proliferation. Scale bar = 100 μm. **E** Scratch-healing assay to evaluate the effect of CHI3L1 knockdown on IEC-6 cell migration. **F** Flow cytometry analysis to assess apoptosis in IEC-6 cells with or without CHI3L1 knockdown. Data are expressed as mean ± SD (*p < 0.05, **p < 0.01, ***p < 0.001, and ****p < 0.0001).

IHC indicated that CHI3L1 was predominantly enriched and expressed in the intestinal epithelial layers of children with NEC. To further investigate the effect of CHI3L1 on NEC, we established an IEC-6 cell line with low CHI3L1 expression using siRNA (knockdown group). Western blot analysis revealed a significant reduction in CHI3L1 protein expression in the knockdown group, which effectively reversed the LPS-induced decrease in ZO-1 protein expression and reduced the levels of IL-1β and IL-6 (Fig. 3A) (*p* < 0.05). Immunofluorescence analysis also demonstrated that CHI3L1 knockdown significantly mitigated LPS-induced loss of the barrier protein ZO-1 caused by LPS treatment (Fig. 3B) (*p* < 0.05). CCK-8 assays confirmed that LPS exposure led to a marked decrease in the viability of IEC-6 cells compared to the nc and knockdown groups (*p* < 0.05). In contrast, CHI3L1 knockdown significantly enhanced cell viability, which was consistent with the results of the EdU assay, showing a considerable increase in the percentage of positive cells and enhanced cell proliferation (Fig. 3C, D) (*p* < 0.05). Subsequent scratch-healing assays indicated that CHI3L1 knockdown alleviated LPS-induced migration inhibition caused by LPS in IEC-6 cells (Fig. 3E) (*p* < 0.05). These findings suggest that CHI3L1 knockdown significantly improved LPS-induced proliferation and migration in IEC-6 cells. Inflammatory factors were also markedly reduced in the CHI3L1 knockdown model. To evaluate the anti-apoptotic effect, flow cytometry was used to assess the percentage of apoptotic cells, revealing that apoptosis in the si+LPS group was significantly lower than that in the nc+ LPS group (Fig. 3F) (*p* < 0.05). Overall, these results indicate that the protective effect of CHI3L1 knockdown on NEC may be linked to its capacity to restore intestinal epithelial cell proliferation and migration and to reduce apoptosis.

### Knockdown of CHI3L1 mitigates excessive autophagy in intestinal epithelial cells through the PI3K-AKT-FoxO1 signalling pathway

**Fig. 4:**
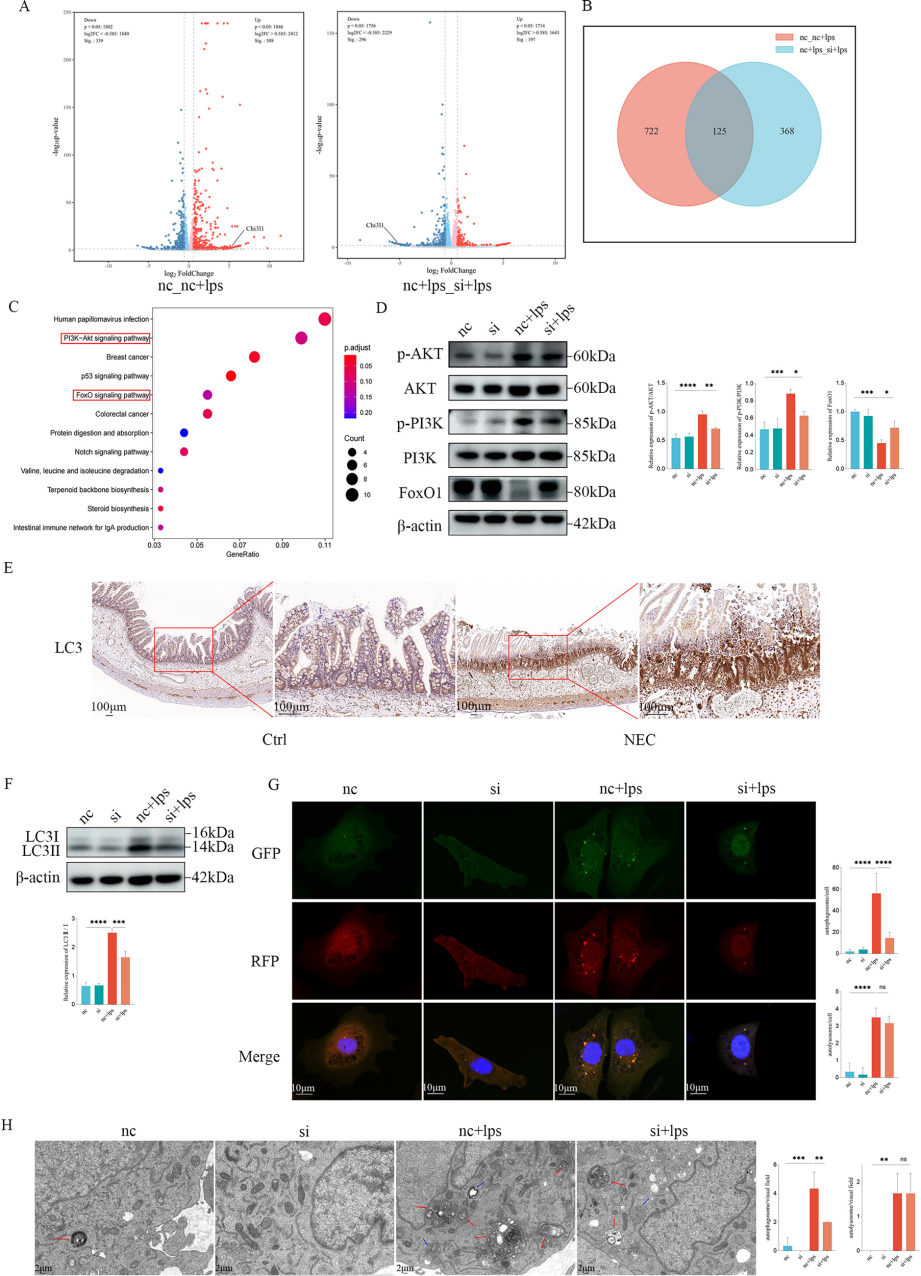
Analysis of changes in the transcriptome and autophagy in IEC-6 cells with or without CHI3L1 knockdown. **A** Differential gene analysis comparing nc, nc+LPS versus nc+LPS, and si+LPS in both groups. **B** Venn plot analysis of nc, nc + LPS versus nc + LPS, and si + LPS in both groups. **C** Differentially enriched KEGG pathways. **D** Western blot analysis showing the expression of p-PI3K, PI3K, p-AKT, AKT, and FoxO1 in different groups of cells. **E** Representative immunohistochemical images of LC3 in the intestines of patients with NEC and controls. Scale bar = 100 μm. **F** Western blot analysis showing the expression of LC3 II/I in different groups of cells. **G** Immunofluorescence analysis of the number of autophagosomes and autolysosomes in different groups of cells. **H** Ultrastructure of IEC-6 cells observed via TEM. Scale bar = 2 μm (Single red arrows indicate autophagosomes and single blue arrows indicate autolysosomes). Data are expressed as mean ± SD (^ns^*p* > 0.05, *p < 0.05, **p < 0.01, ***p < 0.001, and ****p < 0.0001).

Given the beneficial effects of CHI3L1 knockdown on intestinal epithelial cells in NEC, we conducted transcriptome sequencing of three groups of cells (nc, nc + LPS, and si + LPS) to identify the key pathways involved in the anti-NEC effects of CHI3L1 knockdown. We identified 847 DEGs in the nc + LPS group compared with the nc group, with 508 upregulated and 339 downregulated genes. Compared to the nc+LPS group, we found 493 DEGs in the si + LPS group, comprising 197 upregulated and 296 downregulated genes (Fig. 4A). As illustrated in the Venn diagram, 1 215 DEGs were identified when comparing the two groups, and we focused on the rightmost 368 genes for further pathway analysis (Fig. 4B). KEGG enrichment analysis revealed significant enrichment of the PI3K-Akt signalling pathway along with the FoxO signalling pathway (Fig. 4C). To verify whether CHI3L1 knockdown influenced this pathway, we analysed the protein expression levels using western blotting. The results indicated that the levels of p-PI3K and p-AKT significantly increased, whereas FoxO1 levels significantly decreased in the nc+LPS group. However, after CHI3L1 knockdown, the elevation of p-AKT and p-PI3K, as well as the decrease in FoxO1, were reversed (Fig. 4D) (*p* < 0.05). These findings suggest that CHI3L1 knockdown alleviates NEC in vitro by partially inhibiting the activation of the PI3K-AKT pathway and that FoxO1, which is known to be associated with autophagy, plays a critical role in NEC development [18]. Therefore, we hypothesised that CHI3L1 may regulate autophagy through the PI3K-AKT-FoxO1 pathway and performed IHC for the LC3 protein, a key autophagy biomarker, in the small intestinal tissues of children, revealing significant activation of autophagy in NEC (Fig. 4E). Western blot analysis at the cellular level showed that the LC3II/I ratio was significantly increased in the NEC group. In contrast, CHI3L1 knockdown led to a significant decrease in LC3II expression (*p* < 0.05), indicating the inhibition of excessive autophagy activation (Fig. 4F). Furthermore, we utilised the AD-stubRFP-sensGFP-mLC3B adenovirus to monitor the occurrence of autophagosomes and autolysosome in IEC-6 cells. The NEC group exhibited a marked increase in autophagosomes, alongside a rise in autolysosome compared to controls. Following CHI3L1 knockdown, the number of autophagosomes was significantly decreased (*p* < 0.05), whereas autolysosome levels remained unaltered. (Fig. 4G). Using TEM to examine the ultrastructure of IEC6 cells, we observed an increase in autophagosome numbers in the nc+LPS group, while CHI3L1 knockdown inhibited their formation (*p* < 0.05). The number of autolysosomes remained relatively low, aligning with adenovirus immunofluorescence results (Fig. 4H). Collectively, these results suggested that CHI3L1 knockdown inhibits the PI3K-AKT-FoxO1 pathway and alleviates excessive autophagy in intestinal epithelial cells.

### Diagnostic value of serum CHI3L1 in NEC

**Fig. 5:**
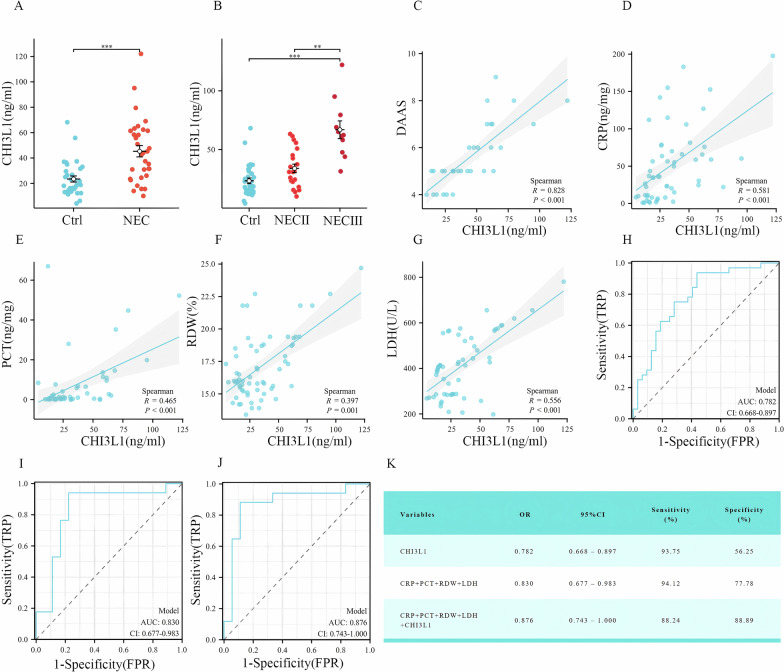
CHI3L1 was analysed at the clinical level. **A** Comparison of CHI3L1 protein expression levels in blood samples from infants with NEC and control infants. **B** Comparison of CHI3L1 protein expression levels in blood samples from infants at different stages of NEC and control infants. **C**–**G** Correlation of CHI3L1 with DAAS, CRP, PCT, RDW, and LDH assessed using Spearman correlation analysis. **H** Diagnostic efficacy of CHI3L1 using logistic regression. **I** Diagnostic efficacy of CRP, PCT, RDW, and LDH using logistic regression. **J** Combined diagnostic efficacy of CHI3L1, CRP, PCT, RDW, and LDH using logistic regression. **K** Statistical parameters of three-group logistic regression. Data are expressed as mean ± SD (**p < 0.01 and ***p < 0.001).

To further validate the clinical significance of CHI3L1 and assess its potential as a specific biomarker for NEC, we examined serum CHI3L1 levels in 32 children diagnosed with NEC and 32 non-NEC neonates matched for gestational age and birth weight. There were no significant differences in the demographic characteristics of the children and the clinical characteristics of the pregnant mothers between the two groups (*p* > 0.05) (Supplementary Material Table 1). Our results indicated that serum CHI3L1 levels were significantly higher in the NEC group than in the non-NEC group, and levels were notably elevated in children with stage III NEC compared with those in the stage II and control groups (Fig. 5A, B) (*p* < 0.05). The Duke Abdominal Assessment Scale (DAAS) is a reliable tool for diagnosing and assessing NEC [19]. Spearman’s correlation analysis demonstrated a strong positive correlation between DAAS scores and CHI3L1 expression levels (R = 0.828, *p* < 0.001) (Fig. 5C). Additionally, serum CHI3L1 levels positively correlated with C-reactive protein (CRP) (R = 0.581, *p* < 0.001) and procalcitonin (PCT) levels (R = 0.465, *p* < 0.001) (Fig. 5D, E). The results showed that CHI3L1 was also significantly positively correlated with red cell distribution width (RDW) (R = 0.397, *p* = 0.001), and lactate dehydrogenase (LDH) (R = 0.556, *p* < 0.001) (Fig. 5F, G). To evaluate the diagnostic efficacy of CHI3L1 and traditional inflammatory markers for NEC identification, we constructed ROC curves for three models: (1) CHI3L1 alone; (2) CRP, PCT, RDW, and LDH combined; and (3) CHI3L1 combined with CRP, PCT, RDW, and LDH. The analysis revealed that CHI3L1 alone demonstrated moderate diagnostic efficacy (AUC = 0.782) (Fig. 5H), while the combination of traditional inflammatory markers CRP, PCT, RDW, and LDH showed improved performance (AUC = 0.830) (Fig. 5I). Notably, the combination of CHI3L1 with these traditional markers achieved the highest diagnostic efficacy (AUC = 0.876) (Fig. 5J), demonstrating superior diagnostic performance compared to the combination of conventional inflammatory markers alone (Fig. 5H–K).

## Discussion

**Fig. 6:**
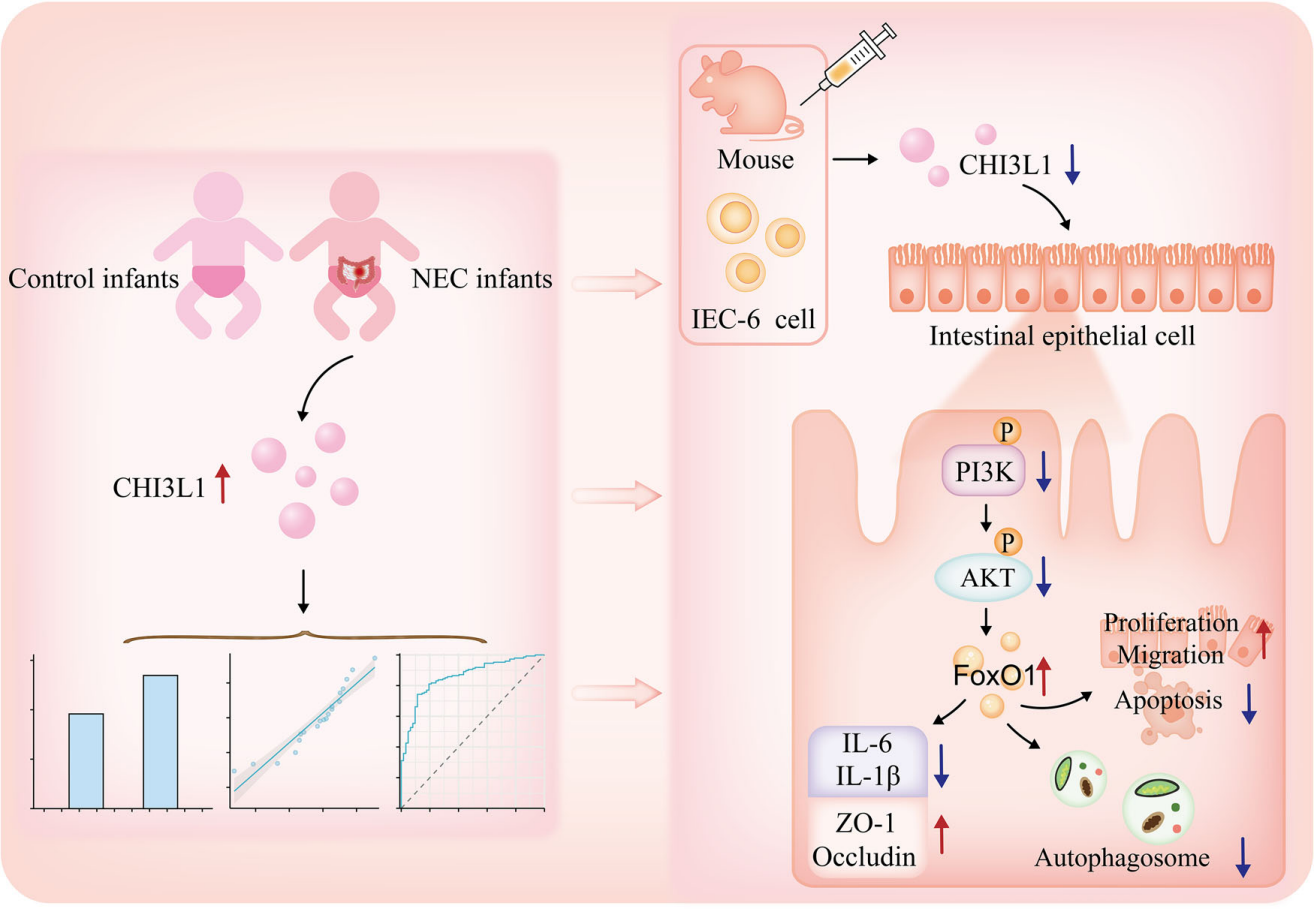
Mechanistic and clinical studies on the possible involvement of CHI3L1 in intestinal epithelial injury in NEC. We identified the key target gene, CHI3L1, which was significantly upregulated in the intestinal tissues of both affected children and model mice from the GEO database. We conducted validations at both the cellular and animal levels, demonstrating that the inhibition or knockdown of CHI3L1 significantly reduced the severity of NEC. Mechanistic investigations revealed that the knockdown of CHI3L1 inhibited the PI3K-Akt-FoxO1 signalling pathway, alleviating excessive autophagy in intestinal epithelial cells, and subsequently reducing injury and inflammatory responses. Moreover, clinical studies indicated that high serum levels of CHI3L1 in children correlate with increased incidence and severity of NEC, as well as with DAAS, CRP, PCT, RDW, and LDH.

In this study, we identified CHI3L1 as a key target in the development of neonatal NEC. A joint intersection analysis of public databases revealed that CHI3L1 is significantly upregulated in NEC, a finding that was further confirmed at the human, animal, and cellular levels. In vitro and ex vivo validations demonstrated that both inhibition and knockdown of CHI3L1 significantly improved NEC-related phenotypes. Further mechanistic investigations indicated that this effect might be mediated through the PI3K-AKT-FoxO1 signalling pathway, which regulates autophagy in intestinal epithelial cells. Collectively, these results suggested that CHI3L1 plays a crucial role in the pathogenesis of NEC. To assess its predictive efficacy for NEC, we examined clinical serum samples and found that CHI3L1 was significantly overexpressed in NEC cases and correlated with disease severity. The potential mechanism by which CHI3L1 is involved in the occurrence and development of NEC is shown in Fig. 6.

CHI3L1 is widely distributed throughout the body and is primarily produced by stellate cells, fibroblast-like cells, endothelial cells, macrophages, and neutrophils [15]. It regulates a variety of pathophysiological processes, including programmed cell death, oxidative damage, activation of the inflammatory response, M2 macrophage differentiation, and Th1/Th2 inflammatory balance, thereby playing a crucial role in inflammation and immune responses as well as tissue injury and repair [15, 16]. CHI3L1 is overexpressed in various inflammatory diseases, such as sepsis, asthma, rheumatoid arthritis, and diabetes [20–22]. We conducted differential gene identification and interaction analysis in the present study using GEO databases across multiple NEC datasets. Our findings revealed that CHI3L1 was significantly overexpressed across all NEC cases, a result that was further validated in intestinal tissues, animal models, and cellular models in children with NEC.

Previous in vitro studies using human colonic epithelial cell lines have demonstrated that inflammatory cytokines, including IL-1β, IL-6, and TNFα, induce high expression of CHI3L1 [23]. In the animal experiments conducted in this study, we found that the upregulation of CHI3L1 levels was positively correlated with the inflammatory markers IL-1β and IL-6, which subsequently contributed to the impairment of the intestinal barrier. These findings suggest a close relationship between elevated CHI3L1 expression and increased levels of inflammatory factors in intestinal tissues.

To the best of our knowledge, no study has specifically linked CHI3L1 to NEC. However, studies on colitis have shown that CHI3L1 enhances bacterial adhesion and invasion in colonic epithelial cells while activating specific cell signalling pathways in intestinal epithelial cells to exacerbate intestinal inflammation [23, 24]. In our study, using the IEC-6 cell line, transcriptome analysis of the CHI3L1 knockdown line indicated that CHI3L1 might target the PI3K-Akt-FoxO1 pathway to regulate the occurrence of NEC. FoxO1 is closely associated with the development of autophagy [25, 26], a key regulator of immune-inflammatory responses and cell death. Previous studies have established the important role of autophagy in the development of NEC in intestinal epithelial cells [27, 28]. Similarly, we found that autophagy was significantly activated in the intestinal tissues of children with NEC. Further analysis revealed an increase in the conversion of LC3I to LC3II in NEC cells, which was reversed by CHI3L1 knockdown. Because autophagy and apoptosis are closely related to cellular self-destructive processes [29], changes in autophagy levels play a crucial role in the fate of the intestinal epithelium in NEC. In this study, we observed that both the levels of autophagy and the degree of apoptosis in intestinal epithelial cells were alleviated to varying extents following CHI3L1 knockdown. Nevertheless, we found a small number of autolysosomes in the in vitro model induced by LPS for 48 h, suggesting that autophagolysosomal function may be impaired in NEC. Importantly, the knockdown of CHI3L1 did not alter the level of autolysosomes but significantly reduced the conversion of LC3I to LCII and the number of autophagosomes, indicating that an increase in autophagosomes along with dysfunctional autolysosomes can lead to the accumulation of damaged organelles and toxic protein aggregates, as well as increased susceptibility to viral and bacterial infections [30, 31]. This can trigger an inflammatory cascade, which is consistent with the mechanisms underlying NEC [27] and aligns with our findings. Therefore, based on our experimental results, we propose that CHI3L1 knockdown does not affect autophagy by altering the lysosomal pathways. Instead, it reduces autophagic flux in the intestinal epithelium by inhibiting the formation of autophagosomes, leading to decreased cellular autophagy and ultimately impacting the outcome of NEC.

The role of CHI3L1 in inflammatory diseases was first identified in patients with rheumatoid arthritis, where serum CHI3L1 levels were significantly elevated during periods of disease activity and were positively correlated with two pro-inflammatory markers, IL-6 and CRP [32]. In addition, serum CHI3L1 levels have been shown to correlate with disease activity in patients with inflammatory bowel disease [33]. To further validate the clinical predictive value of CHI3L1 for NEC, we measured serum samples from children diagnosed with NEC. Our findings revealed that the serum CHI3L1 levels in patients with NEC were significantly higher than those in the control group, with levels increasing further as the condition worsened. In addition, our correlation analysis of serum CHI3L1 and laboratory indicators in preterm infants revealed that serum CHI3L1 levels were positively correlated with CRP, PCT, RDW, and LDH levels. Elevated CRP, PCT, and RDW levels all indicate the presence of infection and a highly active inflammatory response in the body. While CRP and PCT are well-established markers, RDW has been less studied. In critically ill infants with infections, increased RDW levels also suggest excessive inflammatory responses, which may be related to inflammatory cytokines suppressing erythropoiesis and promoting erythrocyte apoptosis through multiple pathways [34, 35]. Elevated serum LDH concentrations indicate increased intestinal permeability, reflecting intestinal injury [36]. This demonstrates that the intensity of systemic inflammatory responses and the degree of intestinal damage are closely associated with serum CHI3L1 levels, though the underlying mechanisms connecting these indicators require further investigation. Furthermore, ROC curve analysis showed that combining serum CHI3L1 with traditional inflammatory markers provides superior predictive efficacy for NEC, suggesting CHI3L1’s potential as a biomarker for NEC prediction. Future multi-centre clinical studies should be conducted to further validate its predictive value for NEC onset.

This study has certain limitations. The human NEC RNA-seq data from NCBI lacked matched gestational age and birth weight between control and NEC groups. To effectively control the impact of this confounding factor on gene expression analysis, we implemented the following dual strategies: First, we identified stable core gene sets through intersection analysis of multiple datasets, then conducted validation using a strictly matched study cohort (1:1 matching for gestational age and birth weight). This approach effectively mitigated the influence of data heterogeneity on research conclusions, ultimately yielding reliable experimental results. Secondly, we did not develop CHI3L1 knockout mice to further elucidate the precise mechanism underlying NEC development using in vivo experiments. In terms of mechanistic investigation, we have not yet explored the specific mechanisms underlying CHI3L1’s interaction with autophagosome maturation. However, these aspects will constitute a primary focus in our subsequent research endeavours.

In conclusion, our study is the first to highlight the critical role of CHI3L1 in the mechanism underlying NEC development. The upregulation of CHI3L1, which is associated with inflammatory mediators, may contribute to excessive autophagy and apoptosis in intestinal epithelial cells through the PI3K-Akt-FoxO1 pathway. We also established a correlation between CHI3L1 levels and clinical NEC by using biospecimen assays. Future multicentre studies with larger sample sizes are warranted to further evaluate the reliability of these findings. Our results provide significant insights into the molecular mechanisms underlying NEC, suggesting that targeting CHI3L1 may be an effective strategy for combating this condition.

## Materials and Methods

### Clinical research and biological sample collection

#### Study objectives and methods

Following approval from the Ethics Committee of the Affiliated Children’s Hospital of Soochow University (Approval No. 2023CS125) and informed consent from the guardians of the patients, we initiated a prospective and dynamic collection of clinical data, laboratory tests, imaging, and biological samples. This collection focused on preterm infants with a gestational age of less than 32 weeks who were admitted to the Department of Neonatology at the Affiliated Children’s Hospital of Soochow University between 1 January 2023 and 30 June 2024. Serum samples were collected at specific time points, starting 24 h post birth and then weekly until the infants were discharged. Infants with severe congenital malformations, a clear diagnosis of inherited metabolic or chromosomal disorders, and from families that declined to participate in the study were excluded.

Preterm infants diagnosed with NEC stage II or higher, based on Bell’s staging criteria [37], were assigned to the NEC group, comprising 32 eligible cases. To ensure comparability, 32 preterm infants hospitalised during the same period were selected as controls, matched for gestational age and birth weight using 1:1 randomisation.

#### Clinical data collection

By reviewing the clinical data extracted from inpatient and outpatient electronic medical records, the collection of electronic medical data primarily included the demographic characteristics of mothers and infants, maternal pregnancy complications, and the general condition of preterm infants, as well as imaging and laboratory examinations. The demographic characteristics mainly encompassed the sex, gestational age, birth weight of preterm infants, and maternal age. Infant conditions covered mode of delivery (caesarean section), multiple births, asphyxia, and small-for-gestational-age status. Maternal factors included pregnancy history, previous miscarriages, premature rupture of membranes, amniotic fluid abnormalities (contamination, polyhydramnios, oligohydramnios), placental disorders (abruption, placenta previa), and pregnancy complications such as diabetes, anaemia, thrombocytopenia, hypertension, severe preeclampsia, eclampsia, intrahepatic cholestasis, hypothyroidism, intrauterine infection, and vaginitis. Laboratory examinations included complete blood counts and biochemical tests, while imaging studies primarily consisted of abdominal ultrasound and abdominal X-ray examinations.

#### Biological samples

The mean time to NEC occurrence in the study was 21 ± 4 d. Serum samples collected within 3 d before the onset of NEC were selected for testing and analysis. Gestational age did not differ significantly between the selected time points in the control group and the time point of NEC occurrence. Intestinal tissue specimens were obtained from patients who underwent surgical treatment for NEC and intestinal atresia. Informed consent was obtained from the child’s guardian before surgery.

#### Reagents and antibodies

The compound 2-({3-[2-(1-cyclohexen-1-yl)ethyl]-6,7-dimethoxy-4-oxo-3, 4-dihydro-2-quinazolinyl}sulfanyl)-N-(4-ethylphenyl)butanamide (K284-6111) was purchased from ChemDiv, Inc. (California, USA). CHI3L1-siRNA (Forward: UGAAGUACCUGAAGAACAATT, Reverse: UUGUUCUUCAGGUACUUC) was purchased from General Biologicals (Hefei, China), with a negative control sequence included. Lipopolysaccharide (LPS) was purchased from Sigma-Aldrich (Darmstadt, Germany). The following antibodies were procured from Cell Signaling Technology (Boston, USA): p-PI3K (17366), PI3K (4292), p-AKT (4060), AKT (4691), IL-1β (63124), and FoxO1 (288). CHI3L1 (ab259322) and Occludin (ab216327) were obtained from Abcam (Cambridge, UK), while CHI3L1 (bs-1093R) was purchased from Bioss (Beijing, China). ZO-1 (21773-1-AP) and LC3 (14600-1-AP) were obtained from Proteintech (Wuhan, China). IL-6 (57315) was purchased from Santa Cruz Biotechnology (San Diego, USA), and β-actin (A17910) was purchased from ABclonal (Wuhan, China).

#### Mice

All experimental procedures in this study were approved by the Ethics Committee of Soochow University (No. SUDA20241008A01) and strictly adhered to national guidelines and regulations. C57BL/6 wild-type mice were obtained from Hangzhou ZiYuan Laboratory Animal Technology Co., Ltd. (Hangzhou, China). Once the mice were successfully modelled, blood samples were obtained via cardiac puncture, and the terminal ileum tissues were harvested through an abdominal incision for further experimental analysis.

#### IEC-6 cell lines

The rat intestinal epithelial (IEC-6) cell line was acquired from Fuheng Biology (Shanghai, China). These cells were routinely maintained in high-glucose Dulbecco’s Modified Eagle Medium supplemented with 10% foetal bovine serum (Gibco, Thermofisher, USA) and 1% penicillin-streptomycin (Gibco, Thermofisher) double antibiotic mixture. The cells were cultured in an incubator with a 5% CO_2_ atmosphere and maintained at a constant temperature of 37 °C.

#### NEC models

NEC was induced in C57BL/6 mice on postnatal day 7, following established methods [38, 39]. The grouping of animals was based on the principle of randomisation. Mice were fed a 2:1 mixture of Similac Advance Infant Formula (Abbott Nutrition, Columbus, USA) and Esbilac canine milk replacer (PetAg). To this diet, lipopolysaccharide (LPS) was added at a concentration of 100 μg/ml, and the formula was administered to the mice at a dosage of 40 μl/g three times daily. In addition, the mice underwent hypoxic stimulation in a chamber with a gas mixture of 95% nitrogen and 5% oxygen for 10 min twice daily, followed by cold stimulation in a 4 °C refrigerator for 30 min after each hypoxic session. The hypertonic formula-feeding regimen combined with hypoxia and cold stimulation was maintained for 96 h. Both the CTRL + K284-611 and NEC + K284-611 groups received daily oral administrations of K284-611 at a dosage of 3 μg/g of body weight [40]. The control group was fed breast milk.

#### Transfection

Lipo3000, acquired from Vazyme (Nanjing, China), was used for transfection, which was conducted in compliance with the manufacturer’s guidelines. The study was organised into four distinct groups: negative control (nc), siRNA-only (si), negative control with LPS (nc+LPS), and siRNA with LPS (si+LPS). Cells were seeded in 6-well plates at a density of 200 000 cells/well and allowed to adhere for 12 h. Subsequently, Lipo3000 and siRNA were combined at concentrations of 7.5 μl/well and 75 pmol/well, respectively, and incubated for 10 min to allow for complex formation. After a 12-h transfection period, the medium was refreshed, and the cells were then challenged with LPS at a final concentration of 20 μg/ml for 48 h. Following transfection and LPS exposure, appropriate assays were performed for detection and analysis.

#### Cell counting kit-8 (CCK-8) assay

After counting, IEC-6 cells (8 000 cells/well) were seeded in 96-well plates. A volume of 100 μL of culture medium was added to each well, and the plates were incubated in a CO_2_ incubator at 37 °C for 12 h. Corresponding siRNAs were then added at a concentration of 3 pmol/well, along with Lipo3000 at 0.3 μL/well, and the incubation continued for another 12 h. Following this, the original medium was discarded, and LPS (20 μg/mL) was added to the corresponding groups. After 48 h of stimulation, the medium was removed, and the CCK-8 working solution was diluted 10-fold. A volume of 100 μl of the diluted CCK-8 solution was added to each well, and the plates were incubated in a 37 °C CO_2_ incubator for 2 h. Finally, the absorbance of each well was measured at a single wavelength of 450 nm.

#### Wound healing assays

IEC-6 cells (2 × 10^5^ cells/well) were seeded in 6-well plates and incubated in a CO_2_ incubator at 37 °C for 12 h. Subsequently, the corresponding siRNAs and negative controls were added according to the different experimental groups, and incubation was continued for another 12 h. Scratches were created in the cell layer using a pipette tip. The plates were washed three times with PBS to remove the detached cells, and LPS was added according to the respective groups. Immediately after scratching, the cell images were captured using a microscope (0 h). After imaging, the plates were returned to the cell culture incubator, and images were captured again 24 h later. The gap area was calculated using ImageJ software.

#### Hematoxylin and eosin (HE) staining

The terminal ileum was collected and fixed in a 4% paraformaldehyde solution for 24 h. After paraffin embedding, the samples were cut into 5 μm-thick sections using a sectioning machine, intestinal damage was assessed after staining with HE, and the histologic lesion score was assessed using a double-blind-observational method [41]. Tissues with a histologic score of ≥2 were considered to exhibit NEC.

#### Immunohistochemistry (IHC)

Paraffin sections were incubated overnight at 4 °C with various primary antibodies, followed by a 1-h incubation at 37 °C with secondary antibodies. Nuclei were stained with haematoxylin and visualised under a light microscope (Olympus, Tokyo, Japan).

#### Immunofluorescence staining

Paraffin sections (5 μm) were de-paraffinised, dehydrated using an alcohol gradient, and subjected to antigen retrieval, followed by rinsing three times at 5 min intervals. The sections were then incubated overnight at 4 °C with various primary antibodies, followed by treatment with secondary antibodies. Nuclei were stained with DAPI, and the slides were visualised under a fluorescence microscope (Olympus, New York, USA).

For confocal microscopy, the cells were seeded in confocal Petri dishes (1 × 10^5^ cells/well) and treated as described in the transfection protocol. The slides were washed with PBS, fixed with 4% formaldehyde, and permeabilised with 1% Triton-X100 for 5 min. Following this, the slides were blocked with 5% goat serum at room temperature for 2 h and incubated with the target antibody overnight at 4 °C. The working solution of the secondary antibody was prepared and added to the corresponding wells (protected from light), incubated at 37 °C for 1 h, and subsequently stained with DAPI. Observations and photographs were taken using a confocal microscope (Olympus, New York, USA).

#### Transmission electron microscopy (TEM)

TEM was used to analyse the ultrastructural characteristics of each group of IEC-6 cells. After fixation, samples were dehydrated and embedded in resin to prepare ultrathin sections. These sections were stained with uranyl acetate and lead citrate to enhance contrast before examination via TEM at the appropriate accelerating voltage. The resulting images were captured and analysed to observe organelles and other fine structures (Hitachi TEM System, Japan).

#### Western blot

The samples were lysed using RIPA lysis buffer, and the lysates were centrifuged after sonication to obtain the supernatant (12000 rpm, 30 min). Equal volumes of protein samples were electrophoresed on a 12–20% precast gel (Genscript, China) and subsequently transferred to a PVDF membrane. The membranes were blocked with skim milk for 2 h at room temperature. Subsequently, the corresponding primary antibody was added and incubated at 4 °C overnight. After three washes with TBST, the secondary antibody was added and incubated for 1 h at room temperature, then washed again three times with TBST. Finally, an ECL luminescence system was employed to visualise the protein bands, which were quantified using ImageJ software. See the supplementary material for original images of the western blot.

#### Quantitative real-time PCR

Total RNA was extracted from the mouse ileum using TRIzol (Vazyme), and its concentration was measured using a Nanodrop spectrophotometer. RNA was then reverse-transcribed to cDNA using a reverse transcription kit (HiScript II Q Select RT SuperMix for qPCR + gDNA wiper; Vazyme). The resulting cDNA product served as a template for qPCR, which was conducted using the AceQ Universal SYBR qPCR Master Mix (Vazyme) on a qPCR detection system (Bio-Rad, California, USA). Relative gene expression was analysed using the 2-ΔΔCT method, with β-actin employed as the internal reference gene. The primer sequences can be found in Table 1 of the main text.

**Table 1:** Primer sequences.

Gene	Forward primer (5′–3′)	Reverse primer (5′–3′)
Mouse IL-6	CTGCAAGAGACTTCCATCCAG	AGTGGTATAGACAGGTCTGTTGG
Mouse IL-1β	GAAATGCCACCTTTTGACAGTG	TGGATGCTCTCATCAGGACAG
Mouse CHI3L1	AGACCCTCCTGTCTGTTGGA	GCCATAAGAACGCAGGAACG
Mouse β-actin	GTGACGTTGACATCCGTAAAGA	GCCGGACTCATCGTACTCC

#### Enzyme-linked immunosorbent assay (ELISA)

Clinical blood samples were centrifuged at 3000 rpm for 20 min, after which the serum was collected. CHI3L1 levels were quantified using the Human YKL-40/CHI3L1/YM1 ELISA Kit (Mlbio).

#### Apoptosis assay via flow cytometry

After the cell model experiment, the cells were stained with 5 μl of Annexin V and 5 μl of 7-AAD for 15 min at room temperature using the BD Pharmingen™ FITC Annexin V Apoptosis Detection Kit I (BD Biosciences). The stained samples were analysed within 1 h using a DxFLEX flow cytometer (BECKMAN COULTER, US).

#### Bioinformatic analysis

Transcriptome data for human and mouse NEC were downloaded from GEO, whereas in vitro model data, including sample preparation, library preparation, sequencing, and analysis, were provided by Tsingke (Shanghai, China). Microarray data were analysed using the Limma R package for differential gene expression analysis, whereas high-throughput sequencing data were processed using the DESeq2 package. Gene set enrichment analysis was conducted in R Studio using the clusterProfiler R package, and ggplot2 was used for visualisation.

#### Statistical analysis

Statistical analyses were conducted using R Studio and GraphPad Prism 9 software. All experiments were repeated at least three times. Descriptive statistics were presented as mean ± SD or median (P25, P75) for continuous variables and frequency (percentage) for categorical variables. For comparisons between groups, the chi-square test was used for categorical measures, whereas the t-test, Kruskal–Wallis test, Wilcoxon rank-sum test, and Mann–Whitney *U* test were used to evaluate the statistical significance of the count data. The survival rate of the model mice was analysed using Kaplan–Meier analysis, and Spearman’s method was used to assess correlations among indicators. Logistic regression analysis was used to examine differences between the NEC group and the control group. A p-value of <0.05 was deemed significant.

## Supplementary information

Supplemental Material-western blot

Supplemental table1

## Data Availability

The datasets generated and/or analysed during the current study are available from the corresponding author on reasonable request.
