# Supplementary material for: Inhibition of CHI3L1 attenuates excessive autophagy in intestinal epithelial cells to reduce the severity of necrotizing enterocolitis
Source: Cell Death Discov. 2025 Apr 5;11:145. doi: 10.1038/s41420-025-02443-7 (PMC11972288; doi:10.1038/s41420-025-02443-7)

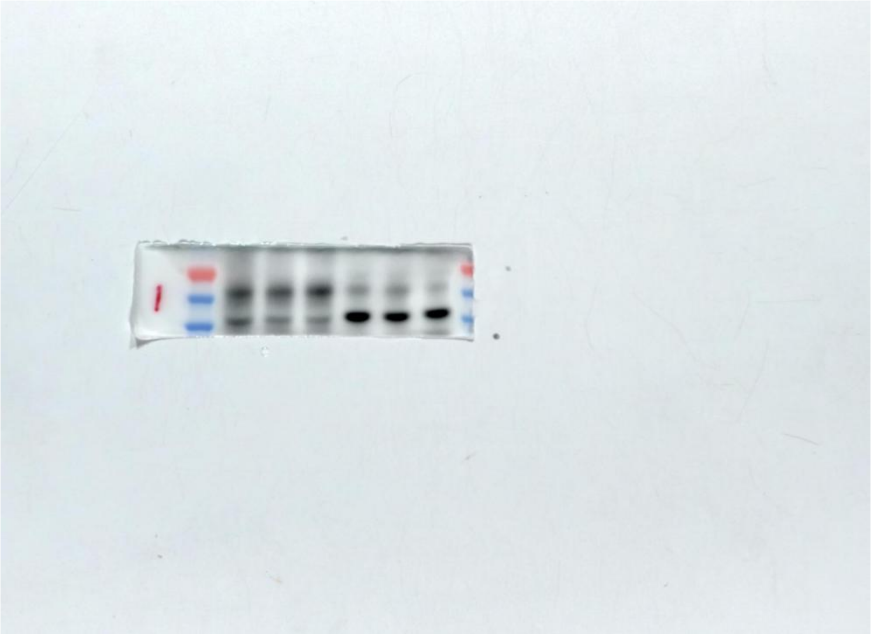

Figure1.D CHI3L1

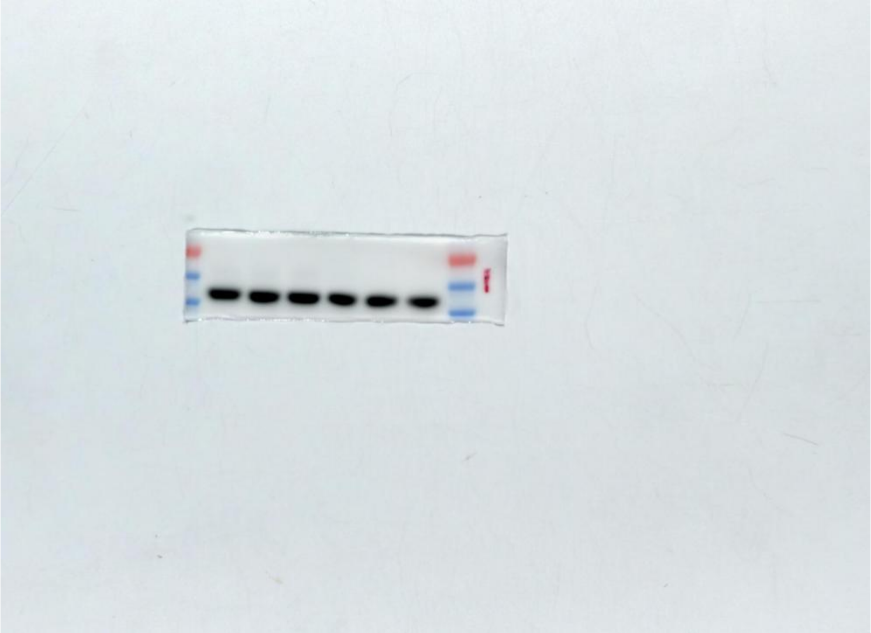

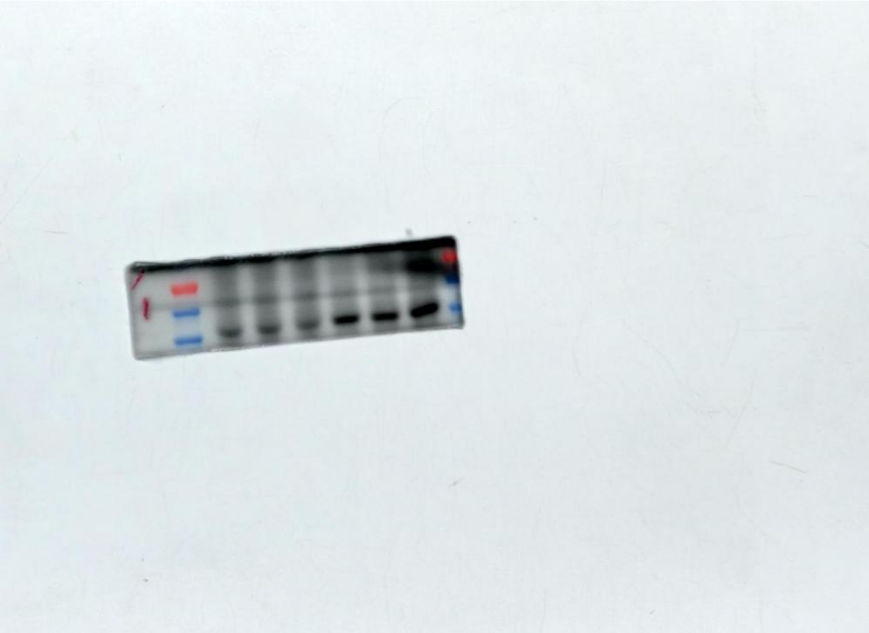

Figure1.G CHI3L1

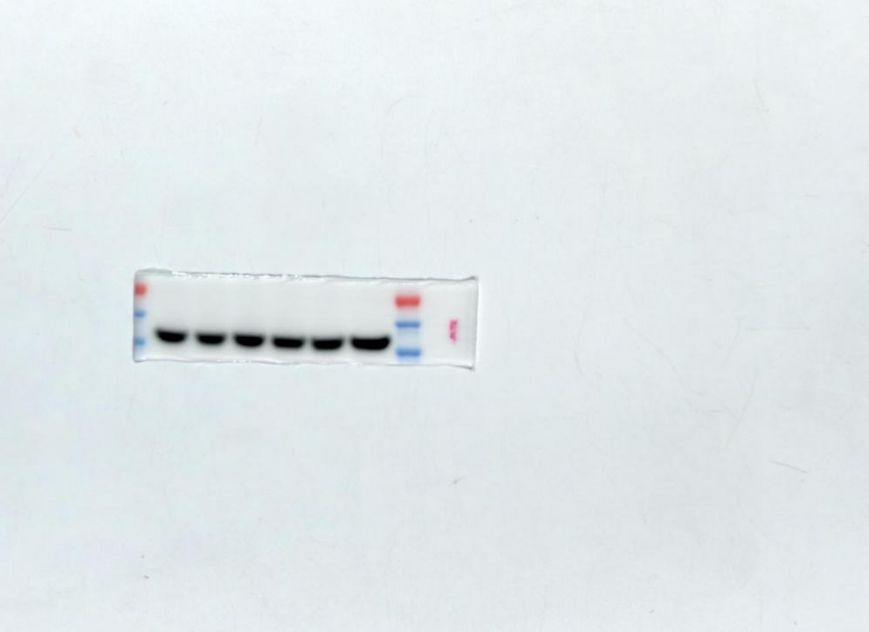

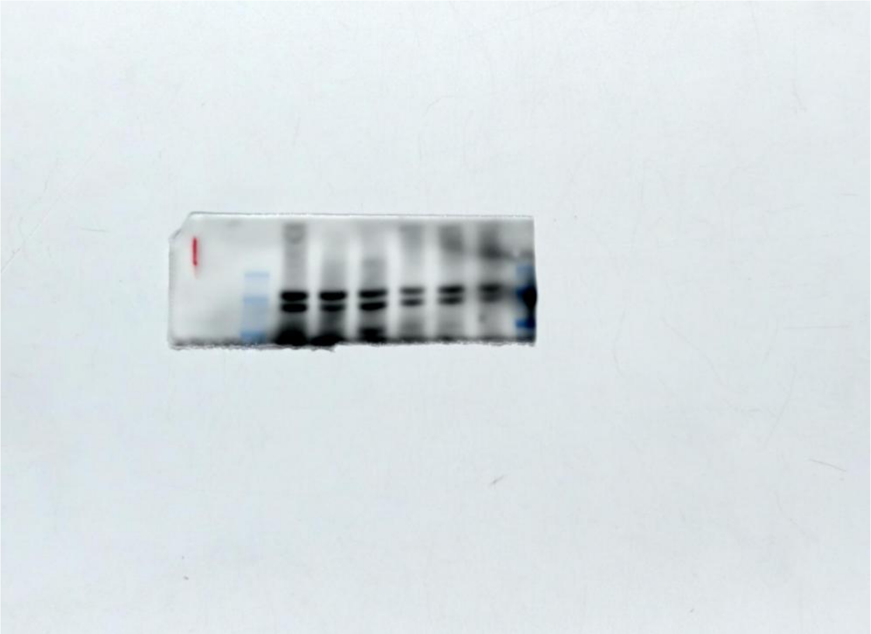

Figure1.G ZO-1

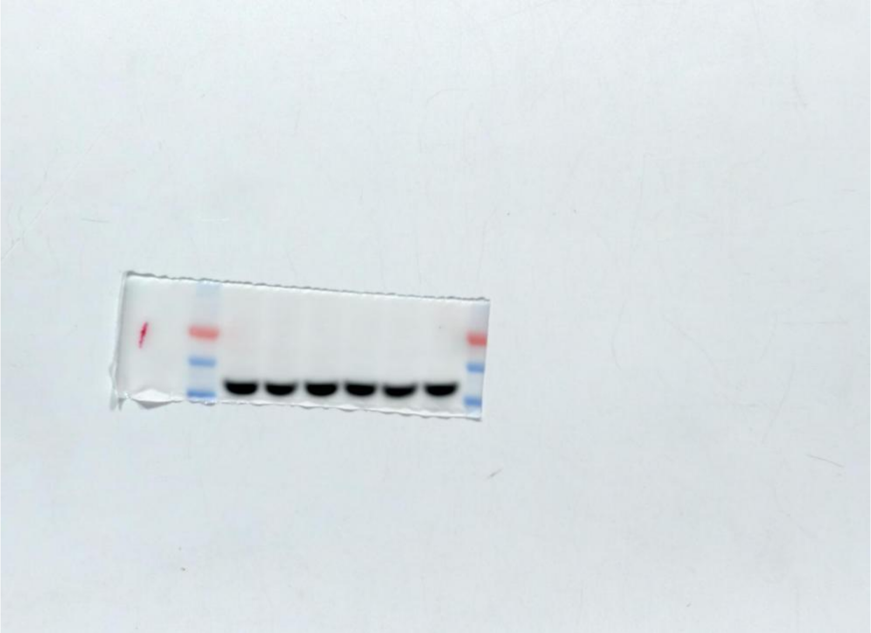

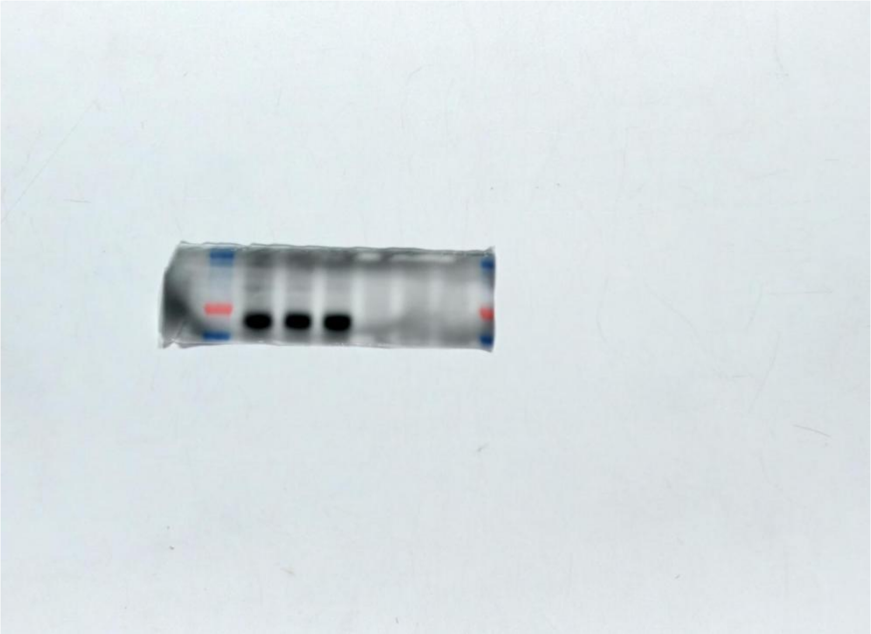

Figure1.G Occludin

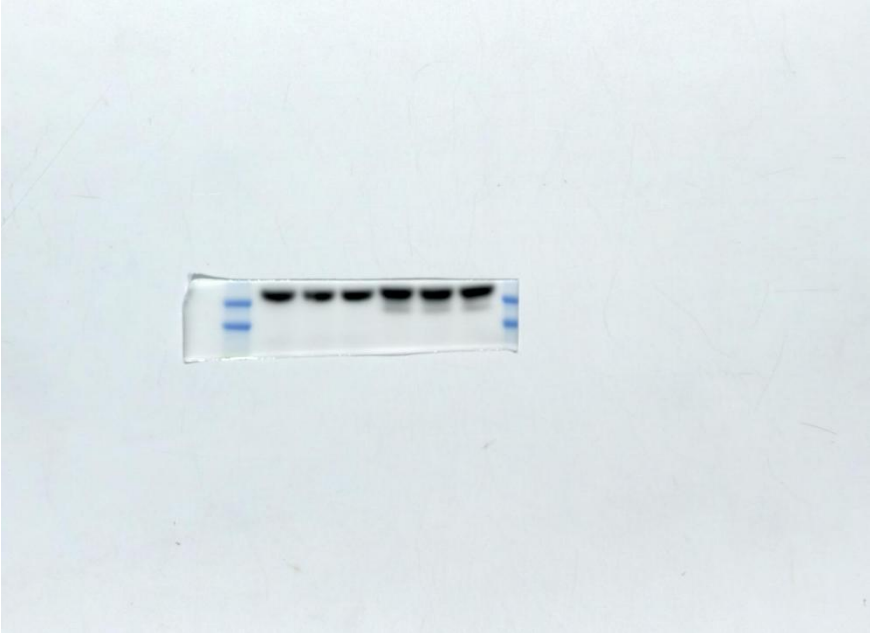

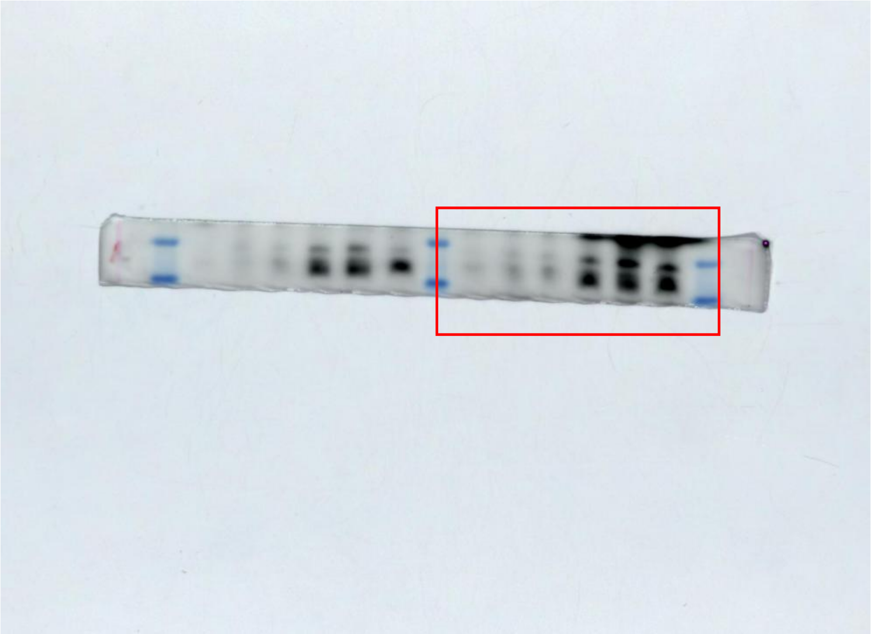

Figure1.G IL-1β

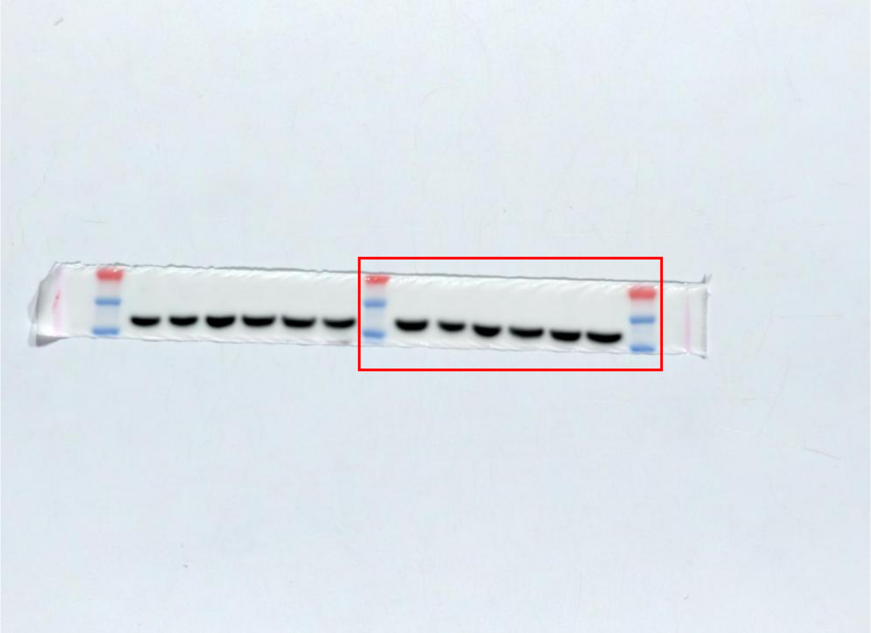

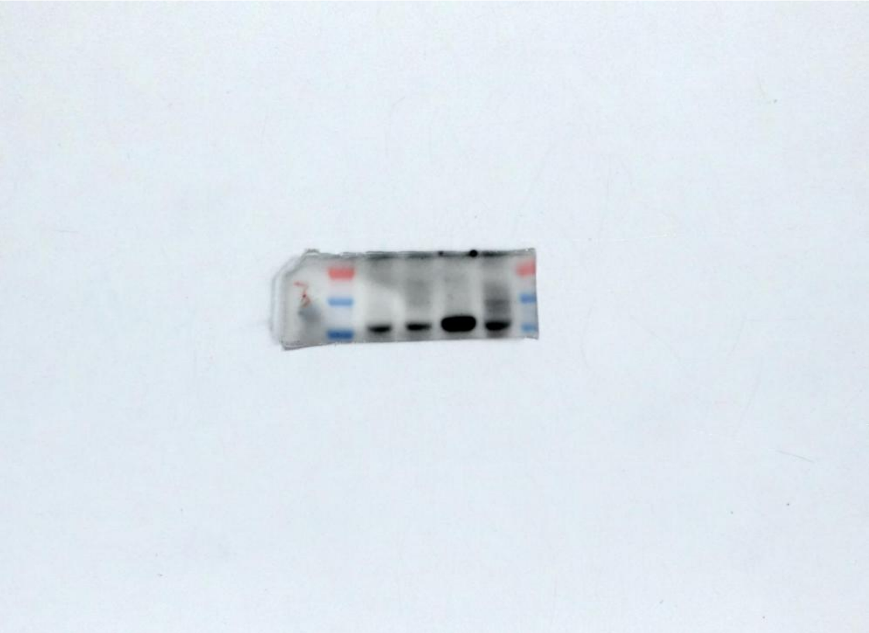

Figure 2.G CHI3L1

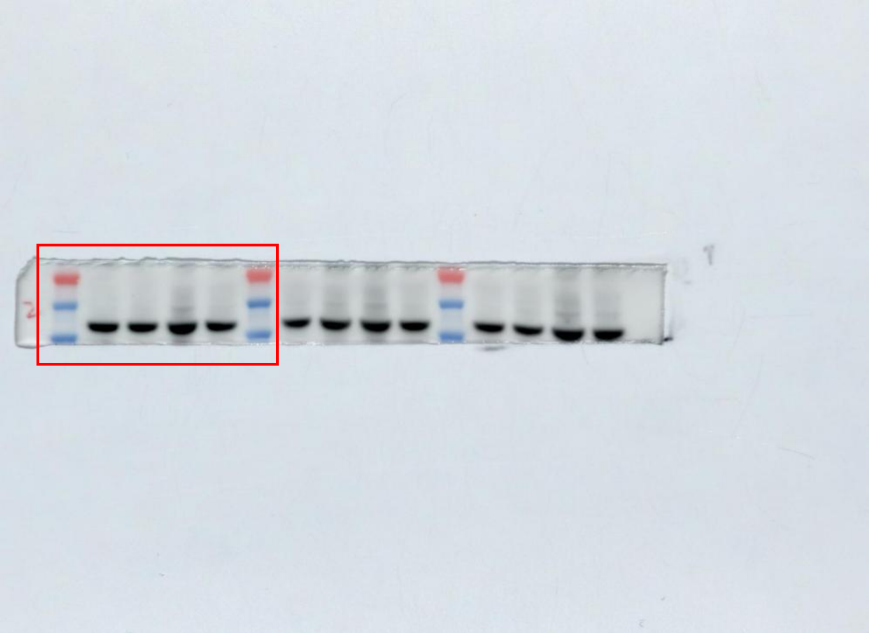

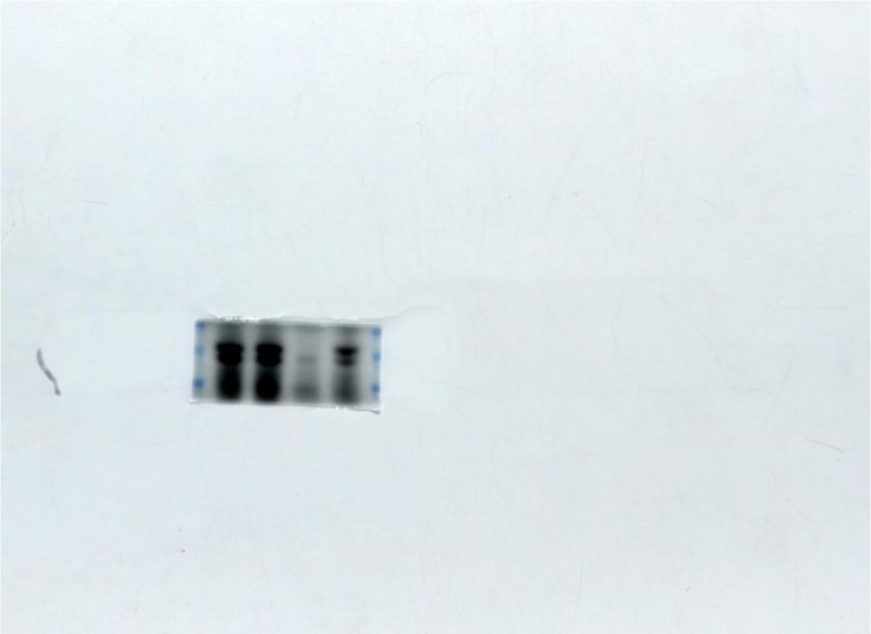

Figure 2.G ZO-1

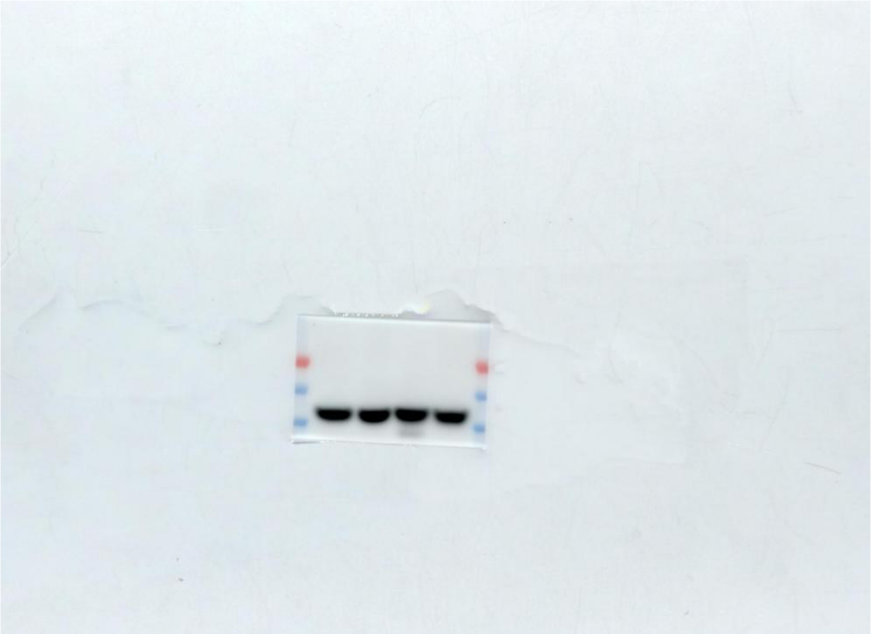

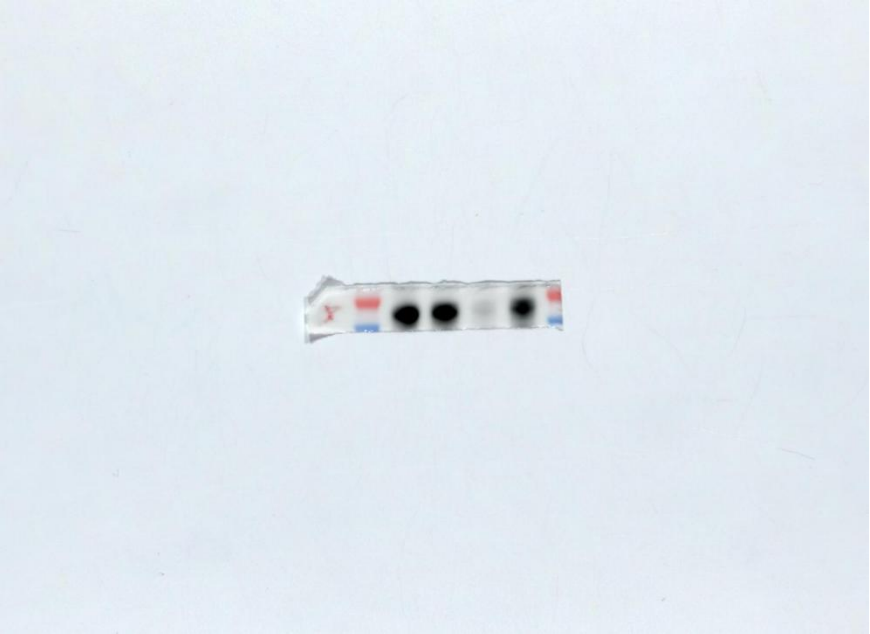

Figure 2.G Occludin

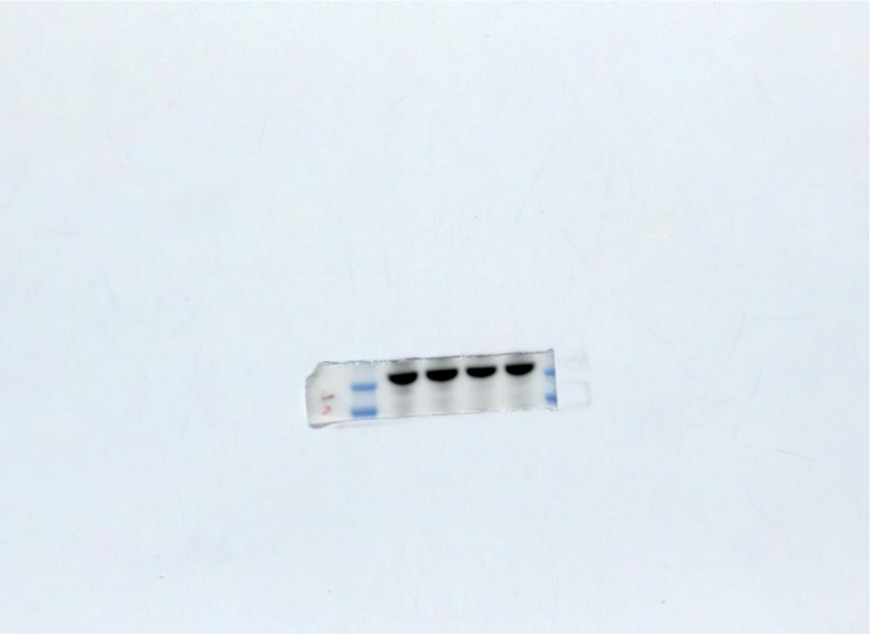

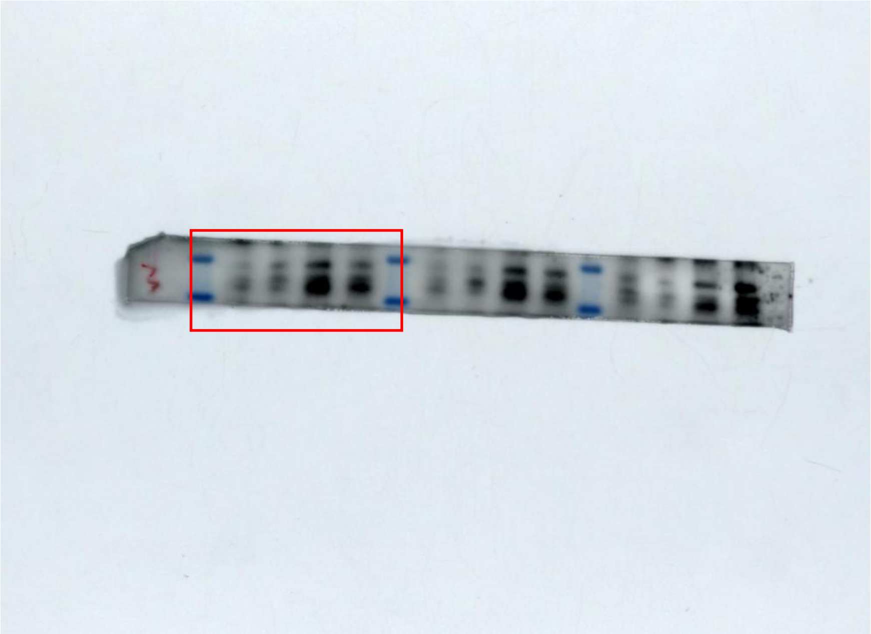

Figure 2.G IL-1β

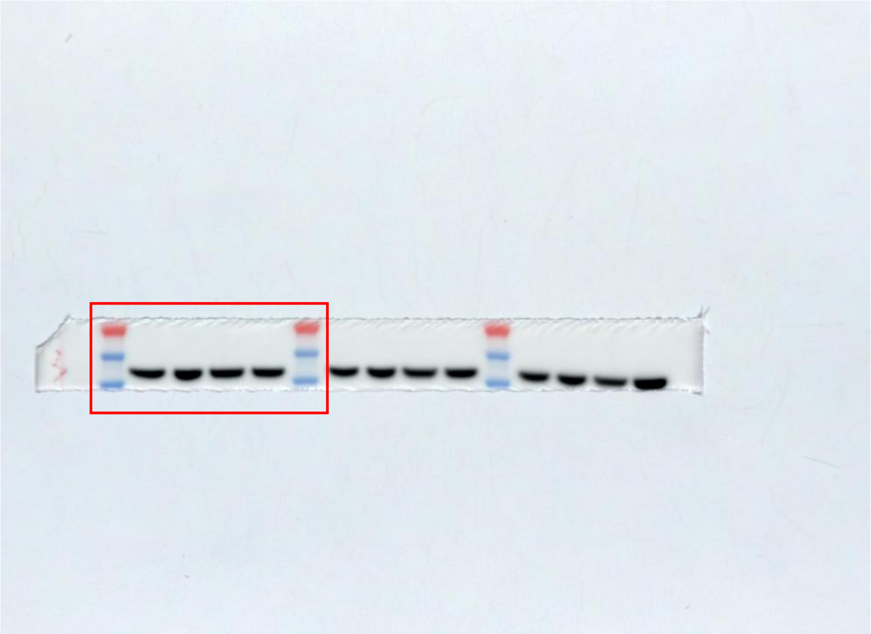

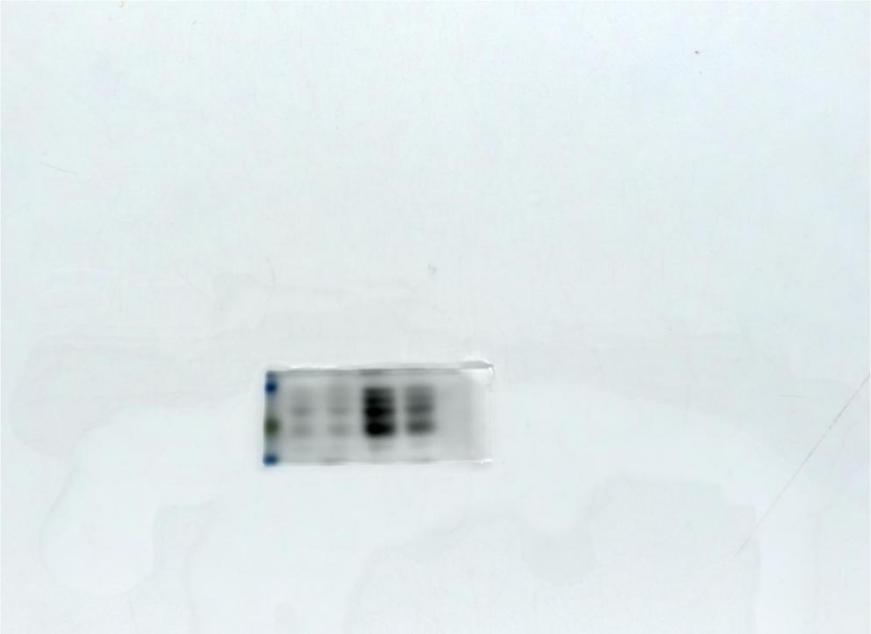

Figure 2.G IL-6

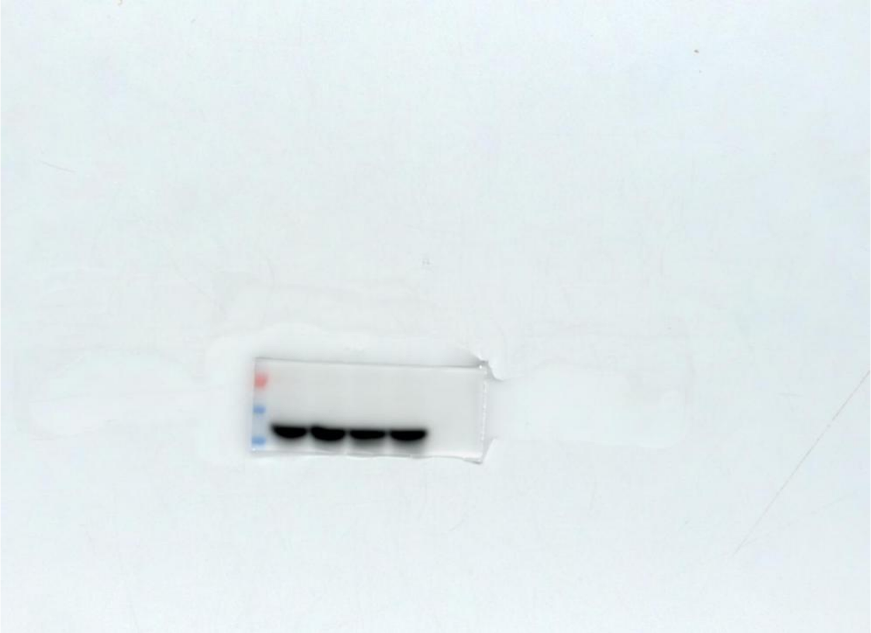

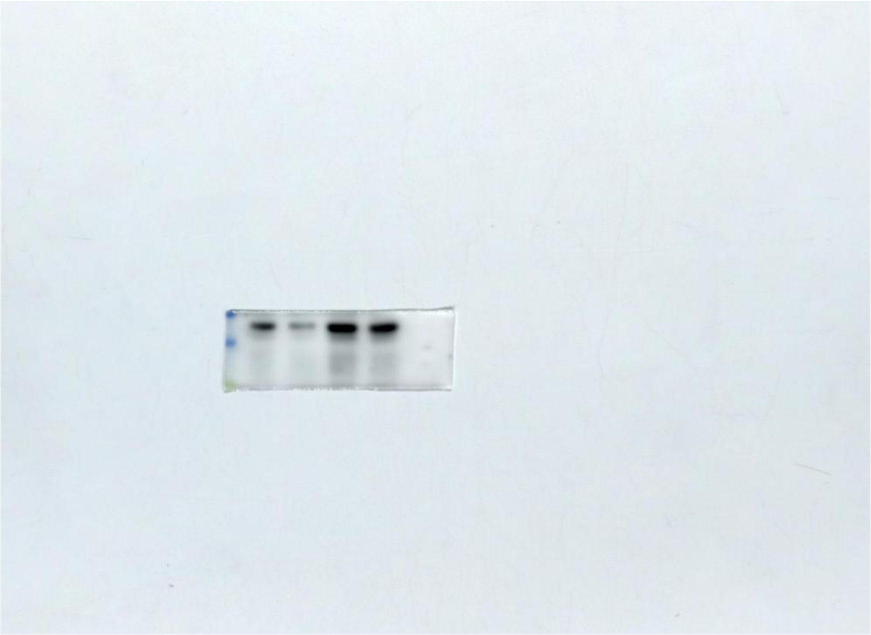

Figure3.A CHI3L1

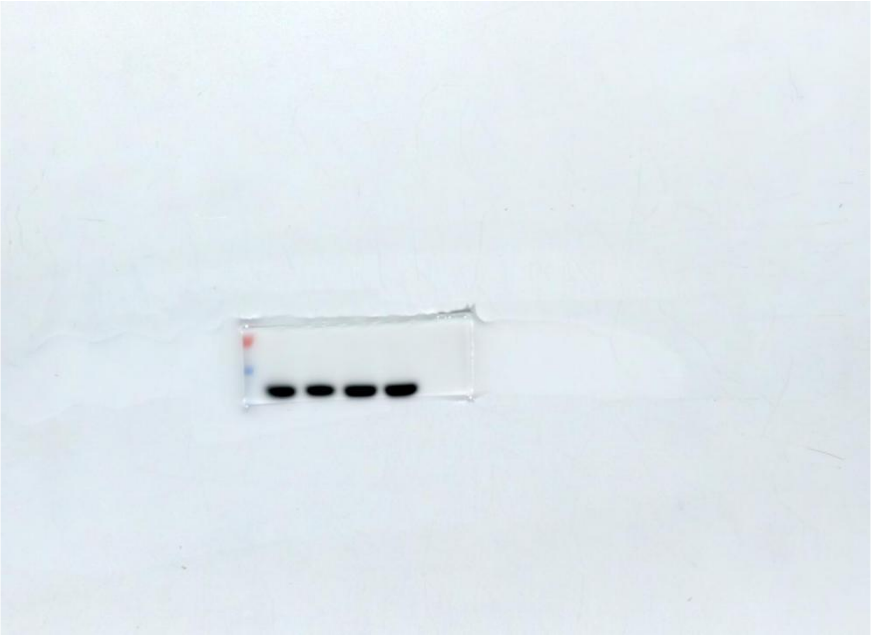

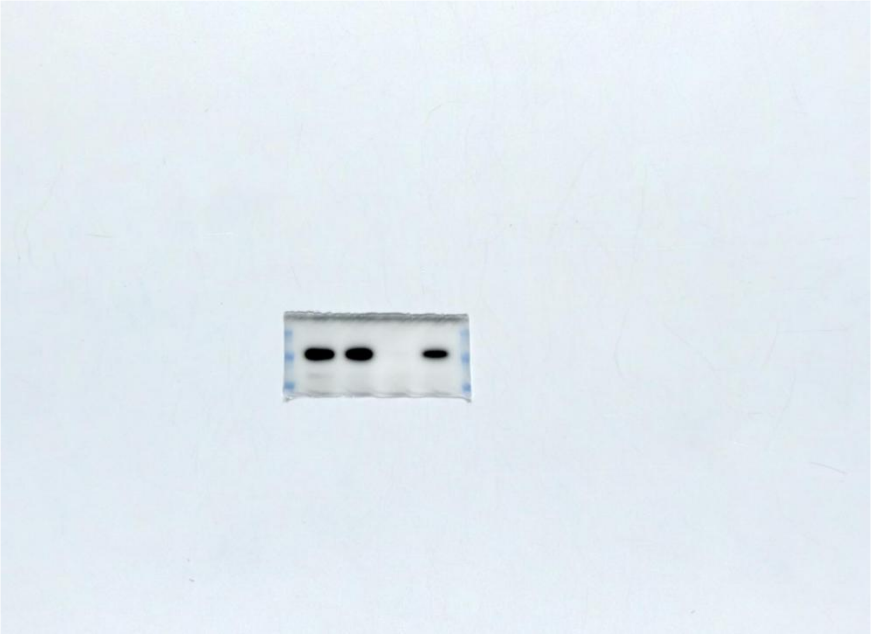

Figure 3.A ZO-1

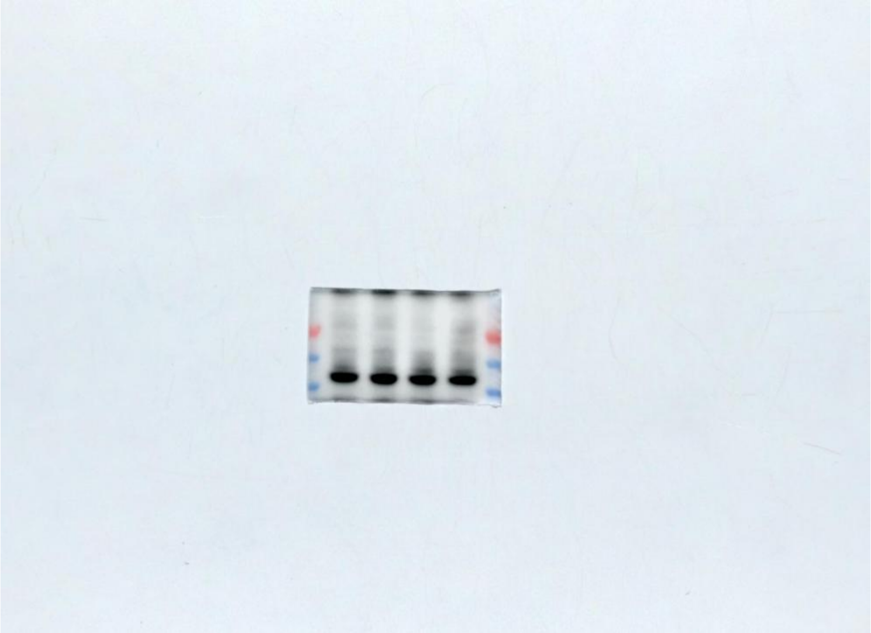

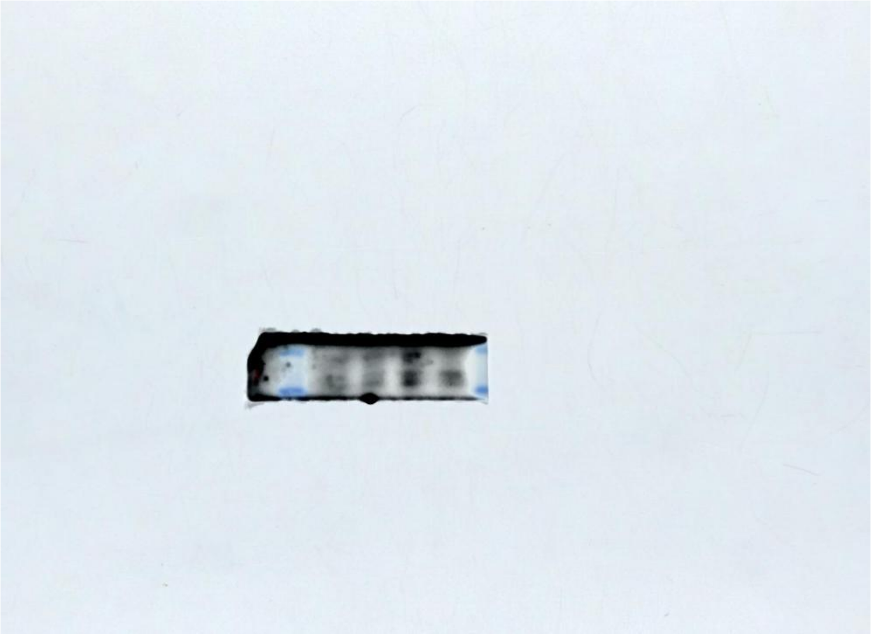

Figure 3.A IL-1β

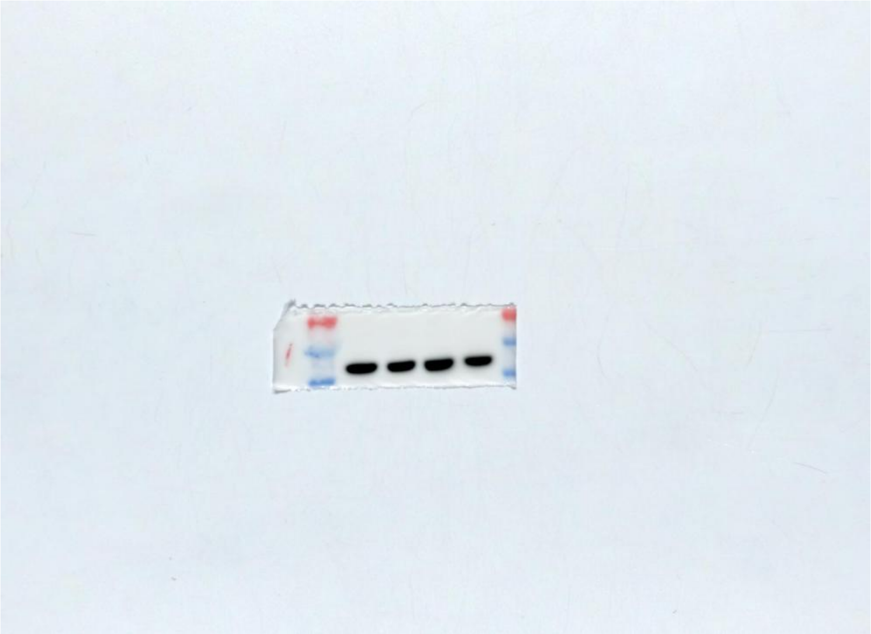

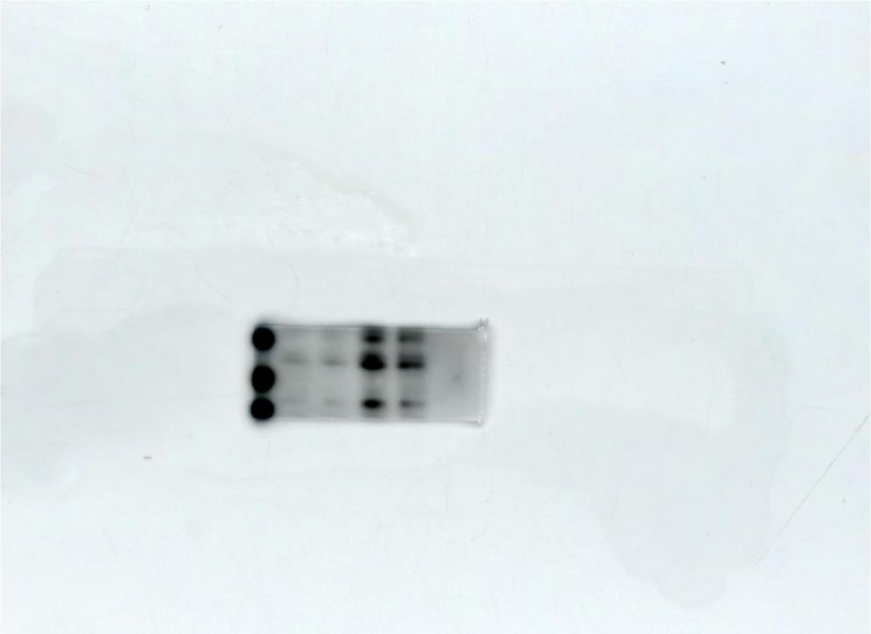

Figure 3.A IL-6

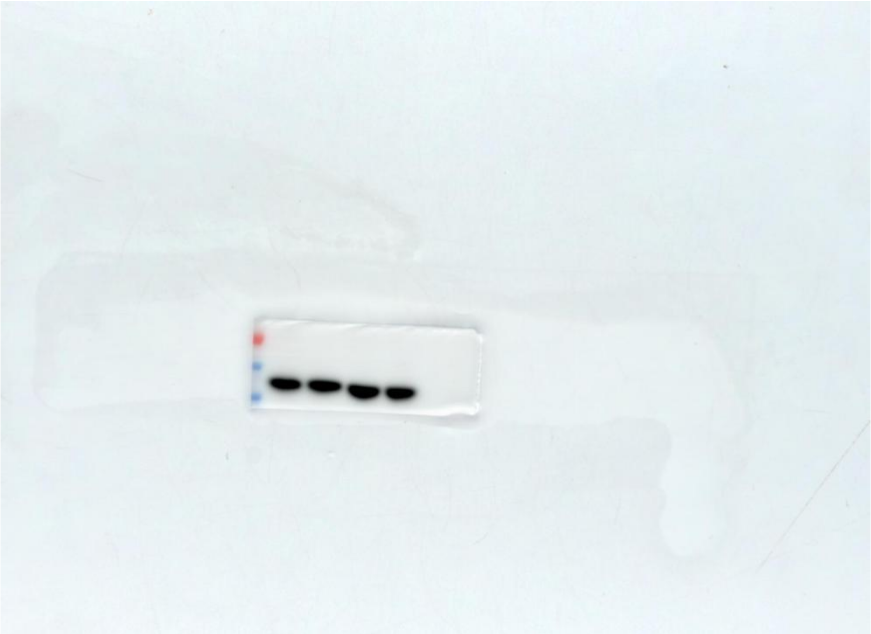

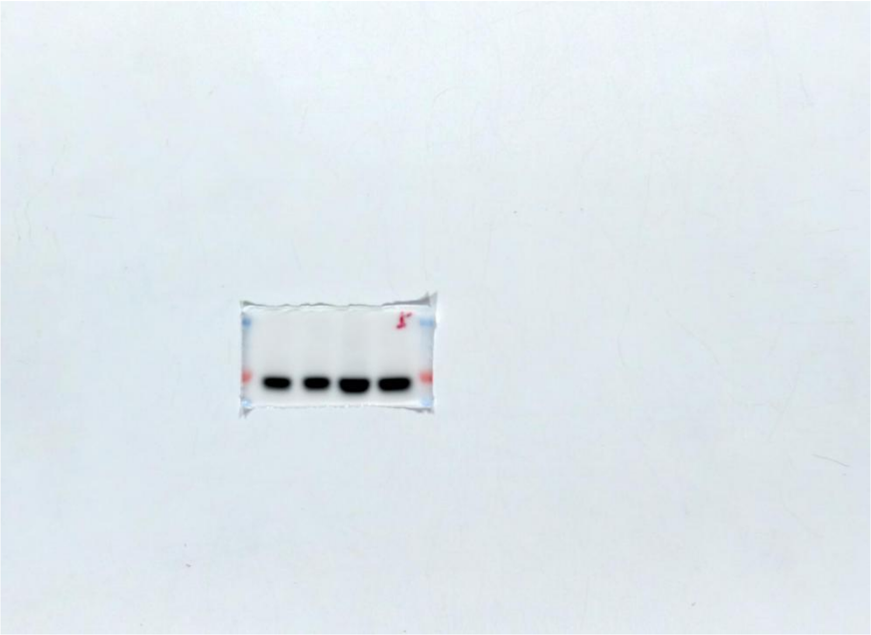

Figure4.D p-AKT/AKT

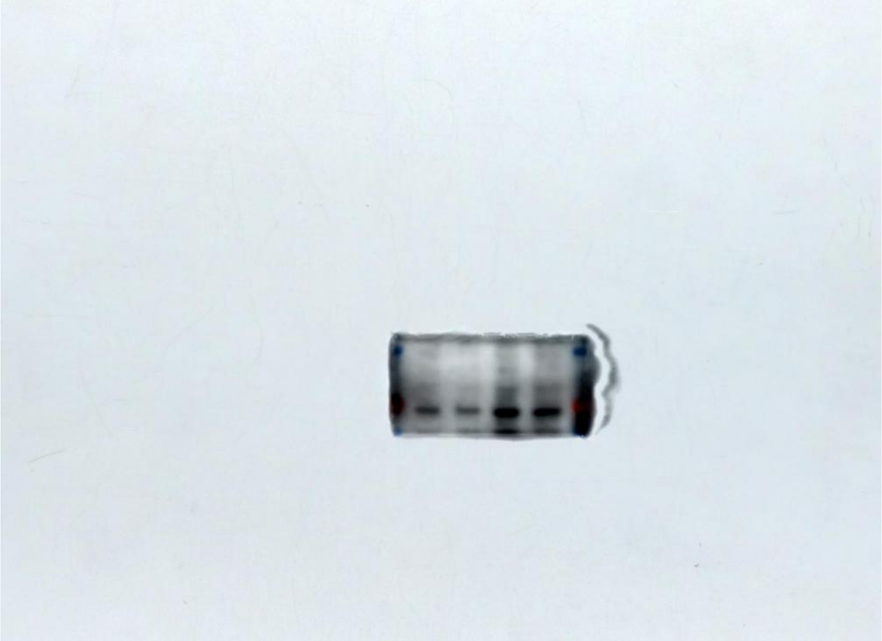

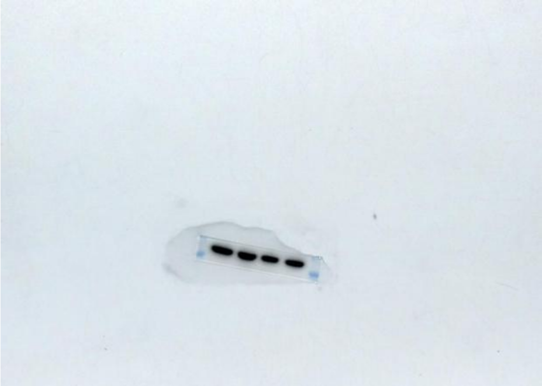

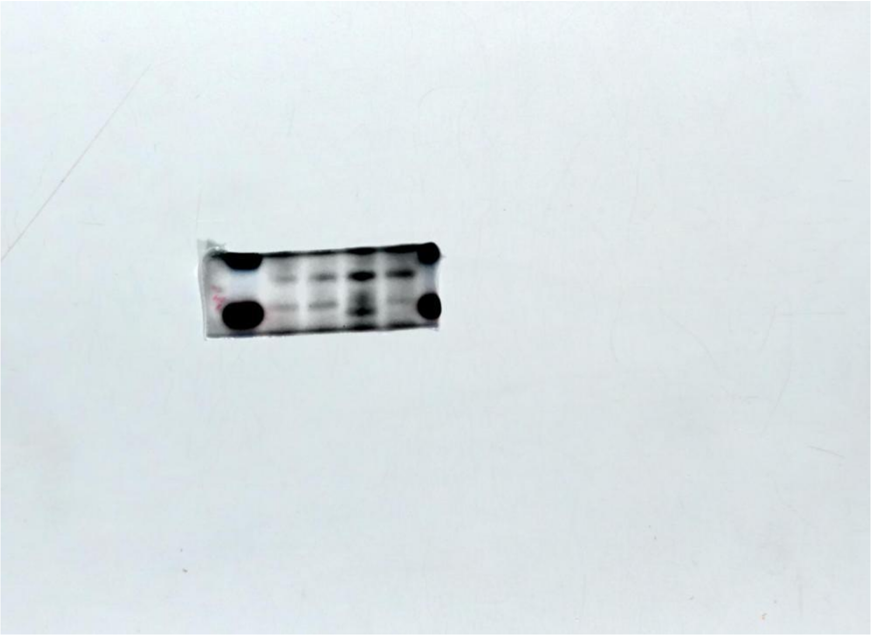

Figure 4.D p-PI3K/PI3K

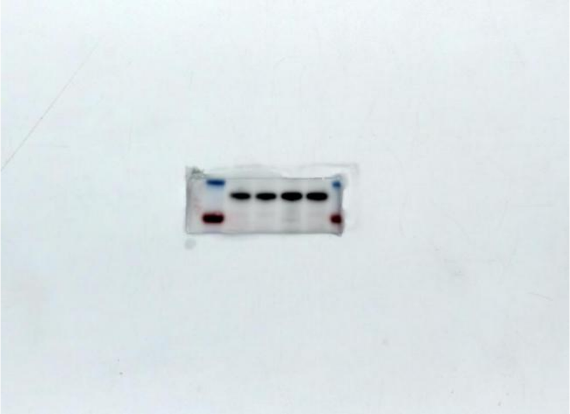

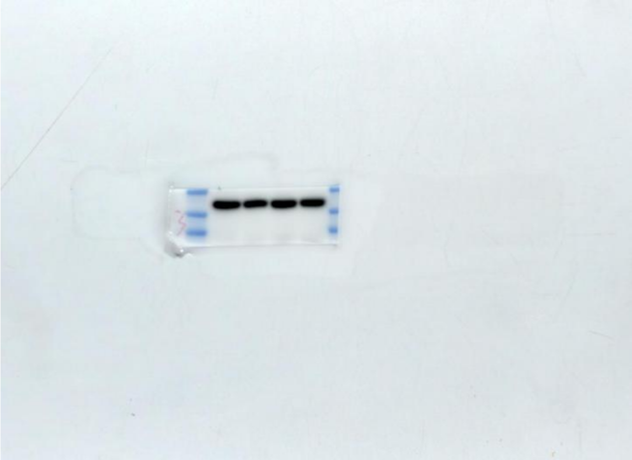

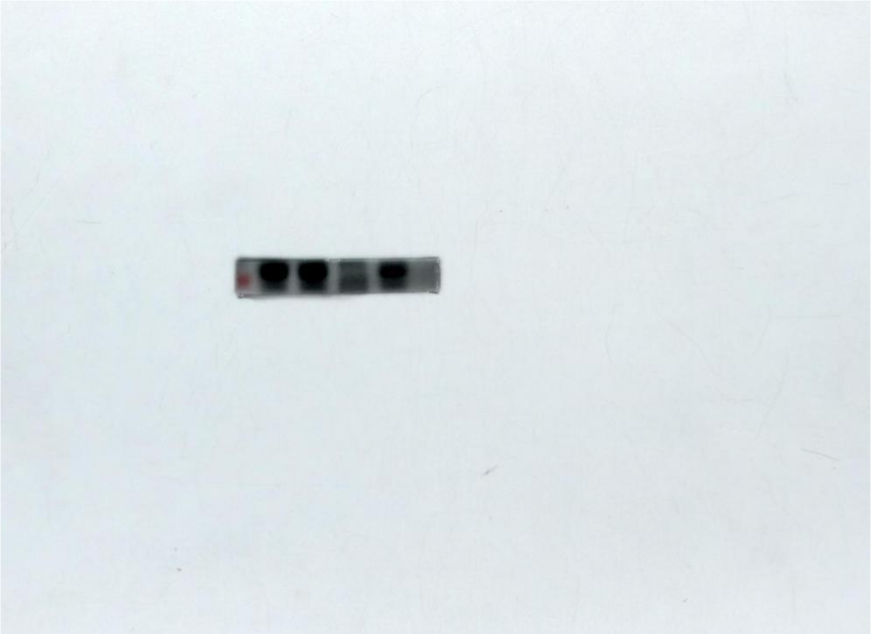

Figure 4.D FoxoO1

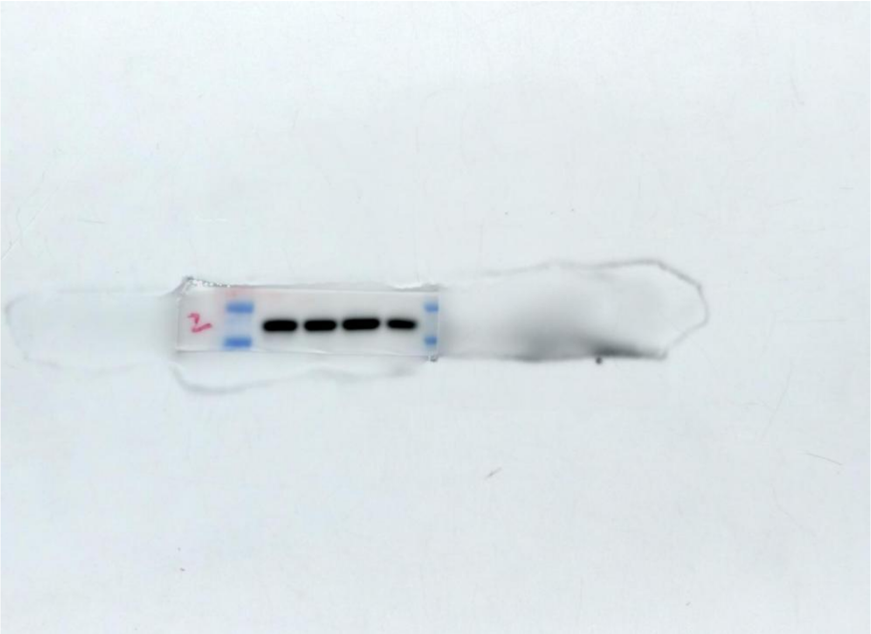

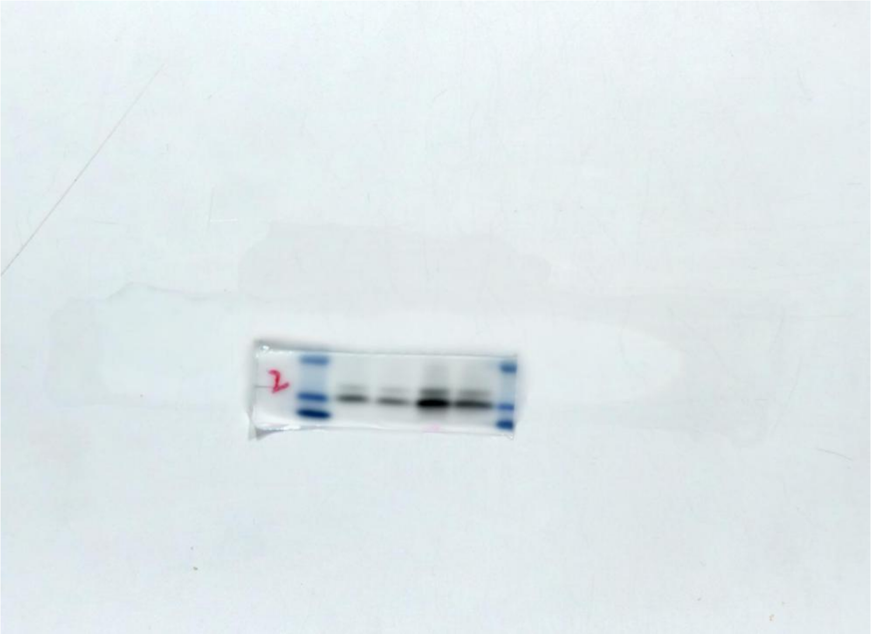

Figure 4.F LC3

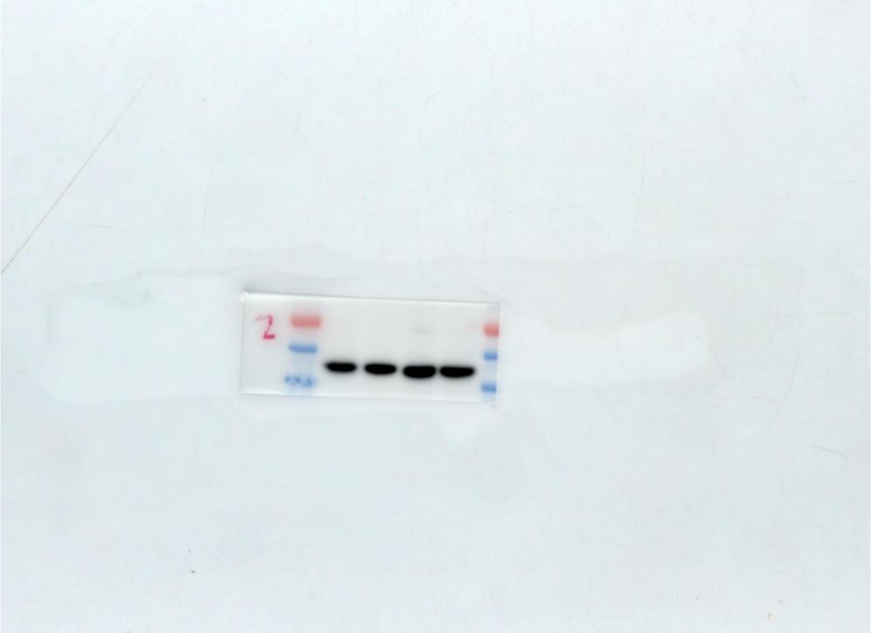

Supplement: Supplementary file 1 — Supplemental Material-western blot [file 41420_2025_2443_MOESM1_ESM.pdf]
