# Supplementary material for: Inhibition of CHI3L1 attenuates excessive autophagy in intestinal epithelial cells to reduce the severity of necrotizing enterocolitis
Source: Cell Death Discov. 2025 Apr 5;11:145. doi: 10.1038/s41420-025-02443-7 (PMC11972288; doi:10.1038/s41420-025-02443-7)
Supplement: Supplementary file 2 — Supplemental table1 [file 41420_2025_2443_MOESM2_ESM.docx]

Table1. Basic clinical characteristics of preterm infants in the necrotizing enterocolitis and control groups

| Considerations | NEC group（n=32） | Control group（n=32） | *P* |
| --- | --- | --- | --- |
| Baseline characteristics of pediatric patients | | | |
| Male（cases，%） | 18(56.25) | 19(59.37) | 0.800 |
| Gestational age[week,（$\bar{X}$±s）] | 28.95±1.93 | 29.15±2.00 | 0.692 |
| Birth weight  [g, （$\bar{X}$±s）] | 1213.81±299.54 | 1232.19±302.08 | 0.809 |
| Cesarean section（cases，%） | 15(46.87) | 15(46.87) | 1.000 |
| In vitro fertilization (cases, %) | 6(18.75) | 4(12.50) | 0.491 |
| Twin or multiple births (cases, %) | 8(25.00) | 4(12.50) | 0.200 |
| Asphyxia (cases, %) | 8(25.00) | 7（21.87） | 0.768 |
| Small for gestational age (cases, %) | 3（9.40） | 2（6.30） | 1.000 |
| Baseline characteristics of the pregnant mother | | | |
| Maternal age[year，M（P25,P75）] | 31（29，34） | 31（28，36） | 0.456 |
| First pregnancy (cases, %) | 12（37.50） | 9（28.12） | 0.424 |
| Miscarriages ≥ 2 (cases, %) | 3（9.37） | 6（18.75） | 0.472 |
| Premature rupture of membranes ≥18 hours (cases, %) | 10（31.25） | 8（25.00） | 0.578 |
| Amniotic Fluid Disorders |  |  |  |
| Amniotic fluid contamination (cases, %) | 4（12.50） | 9（28.12） | 0.120 |
| Excessive amniotic fluid (cases, %) | 1（3.12） | 1（3.12） | 1.000 |
| Low amniotic fluid (cases, %) | 2 (6.25) | 1（3.12） | 1.000 |
| Placental Abnormalities |  |  |  |
| Placental abruption (cases, %) | 1（3.12） | 3（9.37） | 0.606 |
| Placenta praevia (cases, %) | 1（3.12） | 2(6.25) | 1.000 |
| Pregnancy Complications |  |  |  |
| Diabetes (cases, %) | 9（28.12） | 10（31.25） | 0.784 |
| Anemia (cases, %) | 3（9.37） | 4（12.50） | 1.000 |
| Trombocitopenia (cases, %) | 2（6.25） | 0（0.00） | 0.472 |
| Hypertension(cases, %) | 5（15.62） | 2（6.25） | 0.230 |
| Severe Preeclampsia（cases，%） | 5（15.62） | 6（18.75） | 0.740 |
| Eclampsia（cases，%） | 2（6.25） | 0（0.00） | 0.472 |
| Intrahepatic Cholestasis（cases，%） | 1（3.12） | 1（3.12） | 1.000 |
| Hypothyroidism（cases，%） | 4（12.50） | 5（15.62） | 1.000 |
| Intrauterine Infection（cases，%） | 2（6.25） | 5（15.62） | 0.423 |
| Vaginitis（cases，%） | 3（9.37） | 5（15.62） | 0.705 |
